# How are missing data in covariates handled in observational time-to-event studies in oncology? A systematic review

**DOI:** 10.1186/s12874-020-01018-7

**Published:** 2020-05-29

**Authors:** Orlagh U. Carroll, Tim P. Morris, Ruth H. Keogh

**Affiliations:** 1 Department of Medical Statistics, London School of Hygiene and Tropical Medicine, Keppel Street, London, UK; 2 MRC Clinical Trials Unit at UCL, 90 High Holborn, London, UK

**Keywords:** Missing data, Time-to-event, Observational studies, Survival, Epidemiology, Oncology, Multiple imputation

## Abstract

**Background:**

Missing data in covariates can result in biased estimates and loss of power to detect associations. It can also lead to other challenges in time-to-event analyses including the handling of time-varying effects of covariates, selection of covariates and their flexible modelling. This review aims to describe how researchers approach time-to-event analyses with missing data.

**Methods:**

Medline and Embase were searched for observational time-to-event studies in oncology published from January 2012 to January 2018. The review focused on proportional hazards models or extended Cox models. We investigated the extent and reporting of missing data and how it was addressed in the analysis. Covariate modelling and selection, and assessment of the proportional hazards assumption were also investigated, alongside the treatment of missing data in these procedures.

**Results:**

148 studies were included. The mean proportion of individuals with missingness in any covariate was 32%. 53% of studies used complete-case analysis, and 22% used multiple imputation. In total, 14% of studies stated an assumption concerning missing data and only 34% stated missingness as a limitation. The proportional hazards assumption was checked in 28% of studies, of which, 17% did not state the assessment method. 58% of 144 multivariable models stated their covariate selection procedure with use of a pre-selected set of covariates being the most popular followed by stepwise methods and univariable analyses. Of 69 studies that included continuous covariates, 81% did not assess the appropriateness of the functional form.

**Conclusion:**

While guidelines for handling missing data in epidemiological studies are in place, this review indicates that few report implementing recommendations in practice. Although missing data are present in many studies, we found that few state clearly how they handled it or the assumptions they have made. Easy-to-implement but potentially biased approaches such as complete-case analysis are most commonly used despite these relying on strong assumptions and where often more appropriate methods should be employed. Authors should be encouraged to follow existing guidelines to address missing data, and increased levels of expectation from journals and editors could be used to improve practice.

## Background

Time-to-event or survival studies focus on the analysis of times to an outcome or event. Missing data in covariates is a problem in many such investigations. It can render estimators biased if applied to the complete-cases or using an ad hoc approach to handling missingness, and a loss of power to detect associations between explanatory variables and times-to-event. The presence of missing data can also lead to further challenges in a survival setting such as the handling of time-varying effects or dealing with time-dependent covariates when values are missing in the covariates in question. Additionally, it can lead to questions about how best to approach the checking of model assumptions, for example, the proportional hazards assumption when using a Cox model. Missing data also brings further challenges not specific to time-to-event scenarios, such as how to address the selection of covariates into a model or the flexible modelling of covariates. Another type of missingness concerning time-to-event scenarios is missing observations of the event time due to patients being censored, for example, due to administrative censoring or loss to follow-up. This is typically addressed in the analyses for right-censored data, making the assumption that the censoring is uninformative. Missingness in the outcome is not assessed in this review which instead focuses on missing data in the explanatory covariates only.

Complete-case analysis is both a simple and popular method for dealing with missing data, which involves restricting the analysis to individuals with no missing data. Other simple approaches involve replacing missing observations in a covariate with the mean, median or modal value or the use of a missing indicator category for categorical covariates. While popular, these methods can be biased, inefficient or underestimate the variance of estimates. Multiple imputation is an increasingly popular method for handling missing data which involves replicating the original dataset multiple times and in each replication replacing the missing values with plausible observations drawn from the posterior predictive distribution [1]. It is typically conducted using the ‘missing at random’ (MAR) assumption [2], which also subsumes ‘missing completely at random’ (MCAR). MCAR means that missingness does not depend on the observed or missing values while MAR means that missingness is conditionally independent of the missing values given those which have been observed. Further methodology has been developed to adapt the use of multiple imputation in a survival setting. White and Royston in 2009 [3] focused on the Cox model and recommend including the Nelson-Aalen estimate and event indicator in the imputation model. Bartlett *et al* in 2015 [4] described an alternative imputation approach suitable for several analysis models including the Cox model and Keogh and Morris (2018) [5] adapted both approaches to handle time-varying covariate effects - that is, non-proportionality of hazards.

In addition to developed methodology, there have been several published guidelines focusing on how to conduct and report an observational study with some recommendations pertinent to reporting with incomplete covariate data, summarised in Table 1. Some guidelines, such as Sterne et al. [6] focus purely on the handling and reporting of missing data while using multiple imputation, whereas STROBE [7*,*^8^] and ROBINS-I [9] focus more generally on reporting of observational studies. Examples of recommendations range from providing detail on eligibility criteria of patients to clearly stating the selection process for the final analysis model to reporting the amount of missingness in each covariate and which method was chosen to deal with the missing observations. Sensitivity analyses are also recommended to investigate plausibility of any assumptions assumed and the robustness of results. These published guidelines aim to introduce transparency as well as replicability of results if another analyst were to conduct the same investigation.

**Table 1:** Summary of recommendations or considerations from STROBE, ROBINS-I and Sterne et al. guidelines

Recommendation	Explanation	STROBE	ROBINS-I	Sterne et al.
**Patient Selection**				
State eligibility criteria	State inclusion and exclusion criteria of study participants, including criteria concerning missing data	✓		✓
Report the number of individuals at each stage of the study	Give reasons for exclusion at each stage	✓		
	Indicate the amount of individuals discarded due to missingness at each stage of the study	✓		✓
	Give consideration to selection bias introduced by exclusion criteria		✓	
	May use a flowchart to summarise	✓		
**Modelling and Covariate Selection**				
Covariates	Detail whether included as continuous or categorical and, if relevant, detail how the quantitative covariate was categorised	✓	✓	
	Consider departures from linearity for continuous covariates and state which transformation, if any, was used	✓	✓	
State analysis model	make it clear which method will be used to model the data	✓	✓	
Covariate Selection	describe the procedure used to reach the final model	✓	✓	
	this includes, but is not restricted to, missing data imputation, transformation of covariates, interactions between covariates or inclusion of covariates for a priori reasons	✓	✓	
Results	Provide unadjusted estimates and the final adjusted model	✓	✓	
	State the number of participants included in unadjusted and adjusted analyses	✓		
**Missing Data**				
Report the number of participants with missing data	Report this for each covariate of interest or the number of complete data for the important covariates	✓		✓
	Give reasons for missing values	✓	✓	✓
	Investigate if there are key differences between those observed and those with missing data - this may be compared across exposure/intervention groups.		✓	✓
*Missing data methods (general)*				
Which method was used to handle missing data?	State clearly the method used	✓	✓	✓
State any missing data assumptions that were made	Such as whether the data are MCAR, MAR or MNAR	✓	✓	✓
Sensitivity analysis	Should investigate robustness of findings	✓	✓	
	Compare method with a complete-case analysis		✓	
	If necessary, assess validity of methods if there are differences	✓	✓	
	Assess plausibility of missing data assumptions		✓	
*Multiple Imputation*				
Give details of the imputation model	State the software used and key settings for imputation model			✓
	State the number of imputations used			✓
	State variables included in imputation model			✓
	State how non-normal or binary covariates were handled			✓
	Were interactions in analysis model included in imputation model?			✓
If a large fraction of data are imputed, compare observed and imputed values				✓
Missing data assumptions	Discuss if variables included in the imputation model make MAR assumption plausible			✓
Sensitivity analyses	Compare MI results with CC results			✓
	Investigate departures from MAR assumption			✓
	If necessary, suggest explanations for why there are differences in results across sensitivity analyses			✓

Time-to-event studies are commonly conducted in oncology with a search for time-to-event or survival studies on Web of Science indicating oncology to be the most popular category at approximately 30% of journal articles. As such, this review focused on studies conducted in any area of oncology. Common scenarios involve assessing the risk factors of patients developing a specific cancer or investigating factors associated with survival post-diagnosis. Proportional hazards models and Cox regression, in particular, continues to be the dominant analysis technique in time-to-event studies. As such, the review focuses on proportional hazards models while allowing for the extension of the Cox model to include time-varying effects.

Given the developed methodology in this field and the detailed recommendations in place, this review aims to:

- understand which methods researchers are using in time-to-event analyses when missing data are present
- assess if methods used are being carried out appropriately and the relevant assumptions stated
- assess how other challenges such as covariate selection, choice of functional forms (i.e. whether the covariate should be included as a linear term or be more flexibly modelled) for continuous covariates and checking of model assumptions are handled, particularly in the presence of missing data.

## Methods

### Databases, search strategy and screening

Medline and Embase databases were searched for studies published between January 2012 and January 2018 to allow time for developed methods and guidelines to be used in practice. The search strategy for observational studies consisted of three main components: oncology, missing data and time-to-event analyses; additional details can be found in Additional file 1.

For inclusion, studies had to use a proportional hazards or an extended Cox model (includes an interaction between a covariate and time) in a cancer setting. The study also had to have a reference to missing data (either ‘complete’ or ‘missing’) in the abstract or in the full-text. Studies involving only competing risks, frailty models, accelerated failure time models or excess hazards in the abstract or full-text were excluded from the review. If the abstract mentioned a time-to-event outcome but did not specify the analysis models used, the paper proceeded to a full-text review. Papers not written in English or which focused on methodology, meta-analyses, validations of previously created models, and primary or secondary trial outcomes were excluded. However, retrospective observational analyses of a trial cohort were included.

### Data extraction

The information extracted focused on two key areas: missing data and features of the time-to-event analysis. The missing data component assessed the sample size used in the study, how much missing data had been discarded, if assumptions about the treatment of missing data in the analysis were stated and how any missing data were handled in the analysis: complete-case analysis, single imputation techniques or multiple imputation. Where multiple imputation was used, the choice of univariate or multivariate imputation was recorded, the number of imputations used and which covariates were included in the imputation model. Online supplementary materials were accessed only when referenced with regards to the handling of missing data in the text. The features of the time-to-event analysis assessed were whether the proportional hazards assumption was investigated, how covariates were selected for model inclusion and the assessment of the functional form (if continuous covariates were included). We also assessed, where relevant, how missing data were treated in the context of these features. In addition, the software used for the analysis was also extracted by searching for ‘Stata’, ‘SAS’, ‘SPSS’, ‘R’ and ‘plus’ (for S-plus and Mplus). Papers which did not mention one of these six programs were then searched for the software used. A detailed list of the information extracted can be found in Additional file 1 which was motivated by the guideline recommendations found in Table 1 and are evaluated in the “Results” section.

A pilot investigation consisting of 10 randomly selected papers was carried out by OUC, TPM and RHK to assess the consistency of data extraction, refine the data extraction checklist and agree on how to extract information when answers were ambiguous. Data extraction was then carried out by OUC.

## Results

**Fig. 1:**
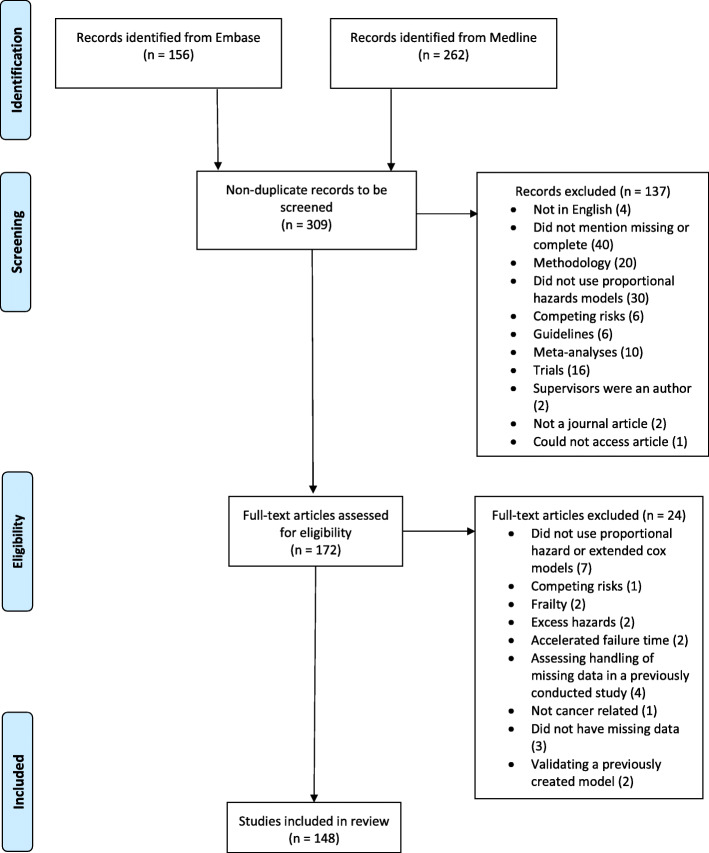
Flowchart of the inclusion process for studies into the review [10]

The PRISM diagram [10] summarising the review inclusion process is shown in Fig. 1. Four hundred and eighteen papers were identified from Embase and Medline, of which 309 were non-duplicates and proceeded to the screening step. One hundred and thirty-seven studies did not meet the inclusion criteria during screening and were therefore excluded. After a full-text assessment, a further 24 studies were excluded with a total of 148 studies included within the review. The studies included came from 110 journals, of which the most prominent were BMC Cancer (5), International Journal of Radiation Oncology, Biology, Physics (4) and Journal of the National Cancer Institute (4).

### Missing data

#### Reporting extent of missing data

In the pilot stage we noticed that many studies excluded individuals due to missing data on key covariates in an initial phase (in which the study population was determined using inclusion or exclusion criteria). That is, having certain covariates observed was used as part of the inclusion criteria. One hundred and six (72%) studies excluded missing data while determining their study population in this initial phase. Of the 106 studies which excluded observations, 66 (62%) reported the number of individuals excluded. On average, 14% of individuals were discarded during this stage. After inclusion criteria had been applied, 102 (69%) studies contained patients with missing data. Table 2 shows the breakdown of missing data during the initial phase and analysis stage of the study.

**Table 2:** Breakdown of the number of individuals with missing data

Description	Number	(%)
**Excluded missing data in initial phase (N=106)**		
Excluded individuals with missing data in any covariate^1^	44	(42)
Excluded individuals with missing data in a subset of covariates	62	(58)
Reported the number of individuals excluded	66	(62)
*Percentage (%) of individuals excluded* (*n*=66)		
Mean (SD)	14.14	(12.40)
Median (IQR)	10.22	(4.73, 18.34)
Min, Max	0.11	47.38
**Missing data present for the analysis stage (N=102)**		
Reported missing data in baseline table for incomplete covariates	82	(80)
Used a complete-case analysis^2^	35	(34)
Used other missing data methods	36	(35)
Quantified the complete-case sample size	25	(25)
*Percentage (%) of individuals excluded* (*n*=25)		
Mean (SD)	31.65	(21.90)
Median (IQR)	31.34	(13.67, 37.76)
Min, Max	1.77	94.16

The initial phase is the stage when defining the study population using inclusion and exclusion criteria.

^1^potentially used a complete-case in initial phase but did not clearly state their methods

^2^A further 31 were not clear on whether they used a complete-case during the analysis

In the demographics table (often considered to be ‘Table 1’ in publications), 87 (59%) studies summarised the missing data in covariates, 47 (32%) reported the breakdown of missingness in incomplete covariates and two (1%) used missing data pattern plots. Thirty-four (23%) used both the text and a table to report the extent of missingness. For the 48 (32%) who did not use a plot, use a table or explicitly break down the missing values in each covariate a general statement was typically made stating which variables were incomplete or that variables or patients were excluded due to having incomplete data.

#### Analyses performed

Table 3 summarises the methods used for the analysis in the presence of missing data. Complete-case analysis was the most popular and was used in 79 (53%) studies either in the initial phase or at the analysis stage (either as the primary method used to deal with missing data or as a sensitivity analysis). This was followed in popularity by removing individuals with missing values in certain key covariates (62, 42%) and multiple imputation (33, 22%). Some studies used multiple methods for handling missing data with 18 (12%) using both complete-case and multiple imputation.

**Table 3:** Methods used in studies for the handling of missing data

Missing data methods	Count	(%)*
Complete-case	79	(53)
Removed individuals with incomplete data for a subset of covariates	67	(45)
Multiple Imputation	33	(22)
Missing indicator	10	(7)
Worst or best case scenario^1^	2	(1)
Stochastic imputation	1	(1)
Mean value imputation	1	(1)
Mode value imputation	1	(1)
Growth models	1	(1)
Bayesian model incorporating handling of missing data	1	(1)
Full-information maximum likelihood estimation ^2^	1	(1)
Selection procedure^3^	1	(1)
Unclear	33	(22)

^*^Percentages do not sum to 100 as there is overlap with some studies using more than one method.

^1^[11*,*^12^]

^2^[11]

^3^A selection model to account for missing data and time-varying covariates [13]

68 (50%) of all studies used a complete-case analysis as their primary analysis method and 24 (16%) reported multiple imputation as their main analysis. Of those using complete-case analysis as the main analysis, nine (13%) also used MI or other methods. Of those using MI as the main analysis, 12 (50%) used complete-case analysis or another method as a secondary analysis.

#### Missing data assumptions

Of the 148 studies, 128 (86%) did not state the assumptions that their chosen analysis made regarding the missing data. Eighteen (12%) stated the MAR assumption, of which 16 (89%) gave a general statement such as ‘MAR was assumed’, with no further explanation. One (0.7*%*) study stated MCAR and another stated ‘missing not at random’.

#### Sensitivity analyses and stating missing data as a limitation

Ninety-eight (66%) studies did not mention the presence of missing data as a limitation to their analysis. Twenty-six (18%) used sensitivity analyses to check the robustness of their final results to either different assumptions concerning the missingness or comparing results with other techniques to handle missing data.

#### Description of complete-case analysis

**Fig. 2:**
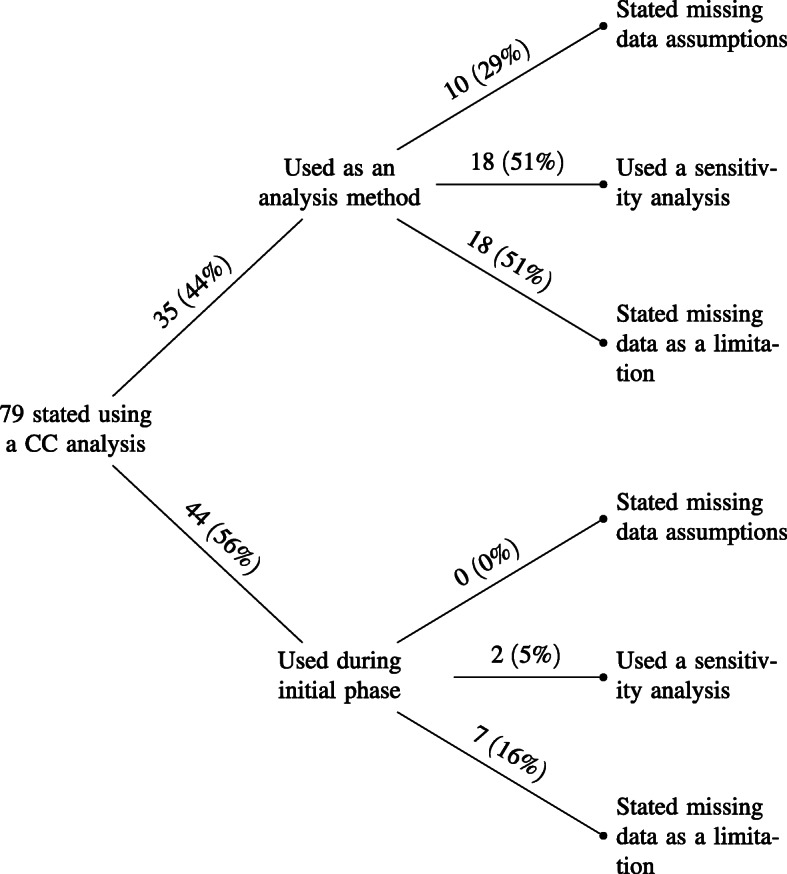
Breakdown of complete-case (CC) usage. The initial phase refers to those who used complete-case analysis when determining inclusion/exclusion of individuals to the study population

Thirty-five (34%) used a complete-case analysis and a further 31 (30%) were suspected to have used complete-case during the analysis stage based on the information provided but did not state this clearly in their paper. On average, 32% of individuals were discarded by applying a complete-case analysis, the maximum being 94% where complete-case was used as a sensitivity analysis for comparison with the main analysis using multiple imputation. Figure 2 summarises the reporting of missing data in the 79 studies that used a complete-case analysis (either during the initial phase or analysis stage). Seven (16%) of the 44 (56%) studies using the initial phase complete-case stated missing data as a limitation. Of the two (5%) studies using a sensitivity analysis, one compared with multiple imputation and the other compared the initial complete-case results pre and post propensity score matching and therefore with different sample sizes. Thirty-five (44%) studies used complete-case during the analysis stage, of which 18 (51%) stated missing data as a limitation. In addition, we presumed based on the information provided that a further 33 studies used a complete-case in the initial phase or analysis stage but did not clearly state this as their method to handle missing data.

**Sensitivity analyses with complete-case:** Eighteen (51%) studies used a sensitivity analysis, of which 14 (78%) involved multiple imputation versus complete-case analysis, two (11%) used complete-case analysis where individuals with missing data in any covariate were excluded versus excluded if there were missing data in a specific subset of covariates (known as available case analysis), one (6%) tested various missing data assumptions and one (6%) did not specify.

#### Description of multiple imputation

**Fig. 3:**
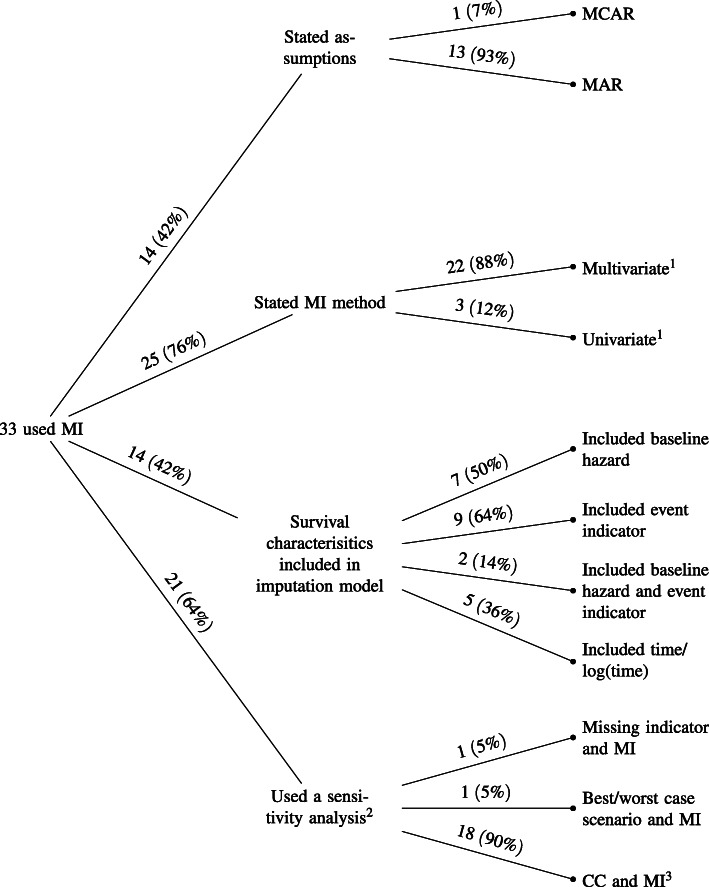
Breakdown of multiple imputation (MI) usage. ^1^ 2 did not specify the type of multivariate MI model used, similarly 1 for univariate. ^2^ 1 study ensured the sample size stayed the same for different models. ^3^ 3 studies did not clearly state that they were using complete-case

The breakdown of multiple imputation usage can be seen in Fig. 3. Thirty-three (22%) studies used multiple imputation, of which 24 (73%) reported the multiple imputation estimates as their main study results. Fourteen (42%) stated a missing data assumption and 25 (76%) described whether a multivariate or univariate approach was taken. For those using a multivariate imputation approach (22, 88%), multivariate imputation by chained equations (MICE) was the most popular method (19, 86%). In total, 14 (42%) studies included a component of the time-to-event outcome in their imputation model. These included the baseline hazard (7, 50%), the event indicator (9, 14%) or both (2, 14%). Twenty-six (79%) studies using multiple imputation stated the number of imputations. One (3%) used a single imputation, five (15%) used five, six (18%) used 10, seven (21%) used 20, two (6%) used 25 and five (15%) used 50. Some studies (example: [14]) cited the White, Royston and Wood paper [15] which suggests that the rule of thumb for choosing the number of imputations should be at a minimum the percentage of cases that are incomplete while other studies (example: [16]) stated the number of imputations with no justification.

##### Sensitivity analyses with multiple imputation:

Of the 21 (64%) studies that conducted a sensitivity analysis, 18 (90%) compared complete-case and multiple imputation (three of which did not explicitly state complete-case) and 10 (56%) used multiple imputation as the main analysis method and one (6%) was unclear on the main strategy while reporting both multiple imputation and complete-case results.

#### Missing data assumptions with methods

Of the 18 studies which stated the MAR assumption, 11 (61%) used multiple imputation, two (11%) used complete-case and one (16%) was not clear on whether they used complete-case or multiple imputation, two (11%) were suspected to have used complete-case but did not clearly state, one (16%) used a stochastic single regression imputation model and one (16%) used a fully Bayesian model. The one study stating MCAR used complete-case analysis and the other stating ‘missing not at random’ performed an analysis using a selection model for the joint distribution of the missing covariates, the outcome and the probability that covariate data are missing [13].

None of the 44 (56%) studies using the initial phase complete-case stated a missing data assumption and for the 35 using complete-case analysis during the analysis stage 10 (29%) stated a missing data assumption. This consists of one (10%) study stating MCAR and nine (90%) MAR, of which seven (78%) used multiple imputation and complete-case analysis together for sensitivity analyses (six (86%) of these used multiple imputation as the main method for handling missing data and complete-case analysis used as a comparison). For the 14 studies using multiple imputation having stated a missing data assumption, 13 (93%) used MAR and one (7%) used MCAR.

### Features of the analysis

#### Selection of covariates into the model

One hundred and forty-four (97%) studies used a multivariable model and therefore used some selection method or criteria to select which covariates should be included. Of these, 85 (59%) stated a clear selection procedure. The use of a predefined set of covariates (33, 39%), stepwise methods (31, 37%) and univariable analyses (32, 38%) were most commonly used, with eight (10%) studies using both a predefined set and univariable analyses. Of the 31 studies using stepwise methods, backwards elimination was used in 18 (58%), six (19%) used forwards selection and seven (23%) did not state which type of stepwise method was used.

Eleven (35%) used complete-case as the main method to handle missing data. Five (45%) out of the 31 studies using stepwise methods stated excluding individuals with missing data on key covariates in the initial phase, and were left with no additional missing data at the analysis phase. Six (55%) studies that used a stepwise procedure did so in a complete-case analysis. We suspect that an additional six (19%) studies used complete-case analysis but this was not clearly stated.

For the 31 studies using stepwise methods, 10 (32%) used them in combination with multiple imputation. Of this, eight (80%) used multiple imputation as the main method to handle missing data, one (10%) used a missing indicator as the main method with multiple imputation as a sensitivity analysis and one (10%) used a sensitivity analysis but did not state whether multiple imputation or complete-case was the main method. For those who used multiple imputation as the primary method to handle missing data, seven (88%) did not state how they combined it with the stepwise methods and the other is suspected to have applied the stepwise procedure in a complete-case analysis to determine the set of covariates to be included, before using this set in the model fitted in each imputed dataset, however this was not clearly stated.

For the 32 studies using univariable analyses, four (13%) studies used multiple imputation as the main analysis method, 14 (44%) stated that they used a complete-case as the main method for handling missing data, 12 (38%) were presumed to have used a complete-case analysis based on the information available, one (3%) used stochastic single regression imputation model and one (3%) did not include incomplete covariates in the analysis model (available case analysis).

#### Functional form of continuous covariates

Sixty-nine (47%) studies included continuous covariates in their model, of which 57 (83%) did not report considering whether any form other than linear was required, i.e. its appropriate functional form. For those that did consider it, splines were the most popular way of transforming covariates in the model (8, 12%), followed by fractional polynomials (2, 3%) and Martingale residuals (2, 3%). Including a quadratic term or a ‘flexible non-linear model’ were each used once. One study used Martingale residuals, cubic splines and fractional polynomials to investigate evidence of non-linear associations [17]. For a further 11 (7%) studies, it was not clear whether included covariates were continuous or categorical. For the 12 studies which reported assessing the functional form of covariates, three (25%) used multiple imputation as the main method for handling missing data, three (25%) used initial phase complete-case, three (25%) presumably used complete-case analysis but this was not clearly stated, one (8%) used stochastic single regression imputation, one (8%) used a study-specific model to impute missing values and one (8%) used available case analysis by restricting to individuals with complete data in the covariates to be included in the analysis model.

#### Proportional hazards assumption and time-varying effects of covariates

The primary analysis method in 142 (96%) studies was the Cox model and the remaining six (4%) stated the use of a proportional hazards model. When investigating the proportional hazards assumption, the covariates included within the analysis model should be assessed. Forty-one (28%) studies stated that the proportional hazards assumption was assessed either using a general statement (example:[18]) or specifically detailing how to handle the covariates which violated it (example:[19]). Of those who checked, seven (17%) did not state the method used to assess the assumption. Schoenfeld residuals were most frequently used (18, 44%), followed by visual inspection of plots of Kaplan-Meier estimates of survivor curves, or functions thereof (12, 29%). Of these two methods, seven (17%) studies used both. Ten (24%) studies tested the assumption by including an interaction between covariates and follow-up time in the model.

For the studies that checked the assumption, 13 (32%) used multiple imputation as the main method to handle missing data, three (7%) used a missing indicator, 11 (27%) used complete-case analysis, seven (17%) presumably used complete-case analysis but did not clearly state, two (5%) had no missing data in covariates chosen for inclusion in the analysis model, one (2%) used both multiple imputation and complete-case but did not state which was the main method, one (2%) excluded incomplete covariates from the analysis model and one (2%) removed individuals with missing data in specific covariates. For the 18 studies using Schoenfeld residuals, six (33%) used multiple imputation as the main method for handling missing data. For the 12 studies using visual inspection of survivor curve plots four (33%) used multiple imputation and for the 10 including an interaction with time, three (30%) used multiple imputation as the main method.

Five studies discovered evidence for time-varying effects, of which three (60%) had incomplete covariates associated with time-varying effects. Two (67%) of these used multiple imputation to impute the missing values in the covariate, of which one took into account the time-varying effect using methods developed by Keogh and Morris [5] and the other stated using MICE while the third study was unclear on how they handled the missing data.

### Software

Forty-four (30%) studies used SPSS, 41 (28%) used SAS, 36 (24%) used Stata, 11 (7%) used R, two (1%) used winbugs, one (1%) used XL-stat life and 17 (11%) did not state. Of these, three (2%) used both SAS and SPSS, one (1%) used SAS and Stata together, one (1%) used SAS and S-plus and one (1%) used SAS and Mplus. Of the 11 studies using R, four (36%) used multiple imputation with three using the MICE package and one using Hmisc. Examples of other potential packages that could have been used are Amelia [20], jomo [21] or smcfcs [22].

## Discussion

Missing data is a pervasive problem in observational time-to-event studies. However, this review has found that few studies appropriately report this issue. Whether this is due to a lack of appreciation of the potential implications of missing data from the researcher, or to the handling of missing data not being deemed of high enough importance to be described in the “Methods” section is unclear. There are general guidelines in place such as STROBE [7*,*^8^] and Sterne’s specific multiple imputation recommendations [6] from 2007 and 2009, respectively, but it appears that many of their recommendations are still not being implemented. By considering literature from 2012 onwards, all papers we reviewed came after the publication of these guidelines. Over half of papers considered (53%) were from 2016 onwards. A surprising finding was that in many studies it was not clear how the study population was selected and what the extent of missing data was. We recommend that authors provide clear and comprehensive information on these aspects including detailing the finalisation of the study population, and stating the sample size used in each model when missing data are present. These recommendations would aid in the transparency of research findings.

Methods for handling missing data such as the multiple imputation approach of White and Royston [3] were implemented by two studies in 2014 [23] and 2016 [24], five and seven years respectively after the method was published. Although valid methods have been developed to handle missing data, the easier-to-implement approach of complete-case analysis is still the most popular method used. However, the studies suggest that little or no consideration is being given to the missing data assumptions needed for this method and whether they are introducing bias to their results. It is plausible that some authors had not noticed the missing data, since software by default runs complete-case analysis without flagging that some individuals were dropped from the analysis. Also of note are the studies which have a ‘fully’ observed dataset and therefore had no need to consider any missing data assumptions or methods. However, this ‘fully’ observed dataset originated from using a complete-case inclusion/exclusion criteria for individuals entering their study. These studies gave no consideration to missing data assumptions and only seven (16%) considered the missing data excluded to be a limitation.

Several systematic reviews have been conducted to assess the handling of missing data in studies, most of which have focus on randomized trials. Wood et al. [25] reviewed the handling of missing outcome data in randomized control trials published in 2001. They found that missing data are typically handled inadequately and that there was almost no use of modern data methods with complete-case used in 46% of studies. Similar findings were made in other reviews covering trials published between 2005 and 2014 [26*–*28]. Karahalios et al. [29] focused on missing data in cohort studies published between 2000 and 2009 and found inconsistent reporting of missing data and inappropriate methods used with 66% of studies using complete-case analysis. With regards to missing data, these reviews collectively looked at studies published between 2001 and 2014. They, along with our own review looking at papers from 2012 to 2018, highlight the lack of progress that has been made in appropriate handling of missing data in both trials and observational studies.

Our review revealed a lack of rigour in other aspects of a study investigation. 42% of studies did not state how the covariates were selected for their final model. When conducting a covariate selection procedure, thought should also be given to continuous covariates and whether categorising is worth a loss of power to detect associations or the occurrence of residual confounding [30]. Clarity should also be required for how the selection procedure is combined when using multiple imputation. For example, [31] states using multiple imputation for multivariable analyses and goes on to detail that univariable models and backward selection were used. However, no discussion is given as to if this process was repeated across the multiple imputed datasets and, if so, what happened when there were disagreements across them regarding the selection process? In 2008, Wood et al. [32] discusses methods to handle covariate selection with multiple imputation. Studies included in the review tended to be exploratory or predictive in nature and consideration should be given to the selection procedure for including covariates into these models. Stepwise methods were used in 37% of studies which stated a covariate selection procedure despite the disadvantages being well-known [33, p. 68]. These include underestimating standard errors of parameter estimates, narrow confidence intervals, low p-values and parameter estimates biased away from zero. VanderWeele also discusses the use of stepwise methods and their drawbacks in a causal setting [34].

For the 41 studies that checked the PH assumption, it was not clear how the 13 studies using multiple imputation incorporated the use of Schoenfeld residuals or inspection of survivor curves as these details were not provided. For those using a time-interaction and multiple imputation, only one did not make it clear how they were incorporating the two methods. Using again the example of [31] it is possible that they checked the assumption using scaled Schoenfeld residuals over time in a complete-case scenario or individually in each imputed dataset but without specification it is difficult to say whether the assumption diagnostics were carried out appropriately. It is important to note that when considering compatibility between the analysis and imputation model thought should also be given to allowing for time-varying effects in the imputation process, in order to allow for valid tests of the proportional hazards assumption. Further thought should also be provided on whether there is sufficient statistical power to detect violations of the proportional hazards assumption [35].

This review demonstrates poor adherence to guidelines already in place and further drives the need for clear reporting. Ideally, an external analyst should be able to rerun the study analysis from the information published which is currently not possible in many studies. Finally, Table 4 provides some related references for consideration of different aspects of missing data and time-to-event features in a study.

**Table 4:** Selected papers describing methods for addressing common issues arising in the analysis of time-to-event data when there is missing covariate data

Consideration	Some recommended references	
**Missing data (general)**		
General recommendations	[6]	*Sterne et al.*: Recommendations for missing data and multiple imputation
Simple imputation	[36]	*Zhang*: Mean, median, mode, regression imputations
Complete-case bias considerations	[37]	*Bartlett et al.*: When CC is valid
	[38]	*Carpenter & Kenward*: When CC is valid
**Multiple imputation**		
Number of imputations to use	[15]	*White et al.*: at least the percentage of incomplete cases
	[39]	*von Hippel*: two-stage quadratic rule
Covariate selection procedures	[32]	*Wood et al.*: Repeated use of Rubin’s rules or stacking approach
	[40]	*Morris et al.*: Adapted for MFP including selection procedure and functional form
Non-linear effects	[40]	*Morris et al.*: Adapted for MFP including selection procedure and functional form
	[41]	*Seaman et al.*: recommend just another variable (JAV) approach
Using a Cox model	[3]	*White & Royston*: inclusion of Nelson-Aalen estimate and event indicator in imputation model
	[4]	*Bartlett & Seaman*: full conditional specification adjusting for the analysis model of choice
Testing the Proportional hazards assumption and modelling time-varying effects of covariates	[5]	*Keogh & Morris*: adapting White & Royston and Bartlett & Seaman approaches for time-varying effects
Time-dependent covariates	[42]	*De Silva et al.*: Investigating performance of two-fold fully conditional specification for time-dependet covariates
	[43]	*Moreno-Betancur et al.*: Use of joint modelling for time-dependent covariates
**Time-to-event features not concerning missing data**		
Functional form	[44]	*Sauerbrei et al.*: multivariable fractional polynomial time i.e. MFP in survival setting accounting for time-varying effects
	[45]	*Buchholz & Sauerbrei*: comparison of procedures for assessing time-varying effects and functional form
	[46]	*Heinzl & Kaider*: Using cubic spline functions to assess functional form
	[47]	*Wynant & Abrahamowicz*: Importance of assessing time-varying effects and functional form
	[48]	*Abrahamowicz & MacKenzie*: Joint estimation of time-varying effects and functional form using splines
Covariate selection procedures	[44]	See above
	[49]	*Yan & Huang*: Assessing time-varying effects using an adaptive lasso method
Testing the Proportional hazards assumption	[35]	*Austin*: Assessing power of tests to assess proportional hazards assumption
	[50]	*Bellera et al.*: Recommend assessing proportional hazards assumption and inclusion of time-varying effects where necessary
	[51]	*Abrahamowicz et al.*: use of regression splines to model time-varying effects
	[52]	*Hess*: use of cubic splines to model time-varying effects
Time-varying effects	[44]	See above
	[45]	See above
	[46]	See above
	[47]	See above
	[48]	See above
	[49]	See above
	[50]	See above
	[52]	See above
**General study considerations**		
Categorising of covariates	[53]	*MacCallum et al.*: Discussion on dichotomising continuous covariates
Non-linear effects	[54]	*Royston & Sauerbrei*: Text book providing overview of model selection with a focus on MFP procedures
	[33]	*Harrell*: Text book providing overview of strategies for regression modelling
Covariate selection procedures	[54]	See above
	[55]	*Heinze et al.*: Review of methods for covariate selection

MFP: Multivariable fractional polynomials

***Limitations of review*** A large number of search terms were used to extract the relevant studies. However, it is possible that some time-to-event studies did not mention how they handled missing data in the title, abstract or keywords and therefore were not included in the review. The search also focused solely on oncology, it is possible that in other medical setting studies could be performed differently in terms of reporting or methods used. A further limitation stems from only one reviewer identifying, screening and extracting information from the studies which may have introduced bias from the selection and interpretation of papers. An agreement check was conducted with RHK and TPM and initially found poor agreement in the collection of sample size of studies and the amount of missing data. The data collection check-list was reviewed and amended to improve discrepancies.

Many journals have a page or word limit which restricts the study analysts from fully detailing methods conducted and results. It is possible that studies were unable to detail information such as checking the PH assessment or conducting a sensitivity analysis. However, most journals also allow for online supplementary materials which could have been used.

For this review we focused on methods used in the oncology field. It is possible that the handling of missing data may be better or worse in other medical fields or study designs.

***Recommendations for multiple imputation in time-to-event analyses*** While it is difficult to recommend a gold standard method as it can depend on the context of the study, for time-to-event studies involving the Cox model we would recommend using the substantive model compatible fully conditional specification (SMC-FCS) of Bartlett et al. [4] as the gold standard method for multiple imputation. It allows for compatibility between the study analysis model and the imputation model. This method is available in both Stata and R software. Keogh and Morris [5] have adapted SMC-FCS to allow for the presence of time-varying effects and proposed an algorithm to allow for model selection with time-varying effects.

White and Royston [3] recommend the inclusion of the event indicator and the Nelson-Aalen estimator in the imputation model for an approximately compatible model. While this is simpler and more straightforward using widely available MI software, the approximation can perform badly in ‘extreme’ scenarios such as strong covariate effects and a high event rate. The approximation also has weaker statistical properties (estimators will generally be inconsistent) than SMC-FCS due to semi-compatibility of the imputation and analysis model. Keogh and Morris have also adapted White and Royston’s method to handle time-varying effects.

## Conclusions

More consideration is required for observational time-to-event analyses with missing data, including clear reporting of how the missing data were handled and how any selection procedures or assumption checks were conducted in conjunction with the missing data method implemented. Wider thought should be given to the limitations the missing data introduces to the observational study, such as bias of parameter estimates, and which methods can be used to help deal with this. While methods such as complete-case analysis are well ingrained in the community there are more modern methods which should also be considered when conducting a study. There appears to be a delay between methodology publication and uptake into the applied research field [56] or, rather, a delay in departing from simpler favoured methods of the field. There are many published guidelines readily available to help researchers conduct and report their study and these should be consulted, alongside a statistician. All recommendations that came from conducting the review were found to have already been emphasised in the published guidance discussed in the Introduction section of this paper. Finally, we recommend that journal editors have requirements for appropriate reporting in the presence of missing data to ensure high quality studies are published and that their results are robust.

## Supplementary information

**Additional file 1** This contains additional information on the database search terms used, the full data extraction checklist and a list of all studies included within the review. This information is stored as a PDF file.

**Additional file 2** This contains the data extraction spreadsheet, stored as a.xls file.

## Data Availability

The dataset supporting the conclusions of this article is included within the Additional files 1 and 2.

## Abbreviations

CC: Complete-case
MCAR: Missing completely at random
MAR: Missing at random
MFP: Multivariable fractional polynomials
MI: Multiple Imputation
MICE: Multivariate imputation by chained equations
