# Supplementary material for: How are missing data in covariates handled in observational time-to-event studies in oncology? A systematic review
Source: BMC Med Res Methodol. 2020 May 29;20:134. doi: 10.1186/s12874-020-01018-7 (PMC7260743; doi:10.1186/s12874-020-01018-7)
Supplement: Supplementary file 1 — Additional file 1 This contains additional information on the database search terms used, the full data extraction checklist and a list of all studies included within the review. This information is stored as a PDF file. [file 12874_2020_1018_MOESM1_ESM.pdf]

1 Search Terms

Table 1: Search Terms used in Medline and Embase databases

#	Terms
1	exp neoplasm/
2	exp RADIATION ONCOLOGY/ or exp MEDICAL ONCOLOGY/ or exp PSYCHO-ONCOLOGY/ or exp SURGICAL ONCOLOGY/
3	tumo?r.mp.
4	cancer.mp.
5	exp pancreas cancer/ or exp female genital tract cancer/ or exp central nervous system cancer/ or exp nasopharynx cancer/ or exp hypopharynx cancer/ or exp cancer antibody/ or exp ovarian cancer cell line/ or exp breast cancer resistance protein/ or exp cancer palliative therapy/ or exp cancer size/ or exp endometrial cancer cell line/ or exp cancer diagnosis/ or exp "HCC cell line (colorectal cancer)"/ or exp cancer staging/ or exp cancer cell/ or exp lung cancer/ or exp experimental pancreatic cancer/ or exp vulva cancer/ or exp uterine cervix cancer/ or exp cancer graft/ or exp cancer immunology/ or exp brain cancer cell line/ or exp colorectal cancer cell line/ or exp germ cell cancer/ or exp larynx cancer/ or exp cancer therapy/ or exp cancer genetics/ or exp skin cancer/ or exp mouth cancer/ or exp cancer statistics/ or exp breast cancer-related lymphedema/ or exp disseminated cancer/ or exp second cancer/ or exp cancer surgery/ or exp childhood cancer/ or exp human epidermal growth factor receptor 2 positive breast cancer/ or exp colon cancer cell line/ or exp esophageal cancer cell line/ or exp cancer survivor/ or exp cancer transplantation/ or exp small cell lung cancer/ or exp pelvis cancer/ or exp cancer adjuvant therapy/ or exp advanced cancer/ or exp lung cancer cell line/ or exp early cancer/ or exp urogenital tract cancer/ or exp rectum cancer/ or exp breast cancer molecular subtype/ or exp metastatic colon cancer/ or exp castration resistant prostate cancer/ or exp triple negative breast cancer/ or exp cancer recurrence/ or exp cancer chemotherapy/ or exp small intestine cancer/ or exp oropharynx cancer/ or exp Institute for Cancer Research mouse/ or exp poorly differentiated thyroid cancer/ or exp non muscle invasive bladder cancer/ or exp differentiated thyroid cancer/ or exp abdominal cancer/ or exp cancer center/ or exp "head and neck cancer"/ or exp ovary cancer/ or exp cancer prognosis/ or exp blood cancer cell line/ or exp "hereditary breast and ovarian cancer syndrome"/ or exp cancer incidence/ or exp cancer pain/ or exp muscle invasive bladder cancer/ or exp biliary tract cancer/ or exp cancer growth factor/ or exp cancer susceptibility/ or exp cancer cell culture/ or exp bladder cancer cell line/ or exp digestive system cancer/

Table 1: Search Terms (continued)

#	Terms
5	or exp cancer control/ or exp nervous system cancer/ or exp hereditary colorectal cancer/ or exp cervical cancer cell line/ or exp childhood cancer survivor/ or exp cancer survival/ or exp liver cancer cell line/ or exp multimodality cancer therapy/ or exp gastric cancer cell line/ or exp inflammatory breast cancer/ or exp brain cancer/ or exp metastatic colorectal cancer/ or cancer*.mp. or exp testis cancer/ or exp gallbladder cancer/ or exp liver cancer/ or exp cancer radiotherapy/ or exp esophagus cancer/ or exp colorectal cancer/ or exp prostate cancer/ or exp uterus cancer/ or exp occult cancer/ or exp cancer registry/ or exp cancer localization/ or exp cancer testis antigen/ or exp heart cancer/ or exp cancer mortality/ or exp breast cancer/ or exp cancer resistance/ or exp cancer specific survival/ or exp cancer tissue/ or exp cancer patient/ or exp cancer associated fibroblast/ or exp cancer screening/ or exp cecum cancer/ or exp cancer vaccine/ or exp adrenal cancer/ or exp respiratory tract cancer/ or exp multiple cancer/ or exp estrogen receptor positive breast cancer/ or exp tongue cancer/ or exp stomach cancer/ or exp cancer grading/ or exp hereditary nonpolyposis colorectal cancer/ or exp pancreatic cancer cell line/ or exp cancer classification/ or exp non melanoma skin cancer/ or exp cancer fatigue/ or exp prostate cancer cell line/ or exp early cancer diagnosis/ or exp colon cancer/ or exp cancer model/ or exp cancer stem cell/ or exp vagina cancer/ or exp penis cancer/ or exp cancer risk/ or exp non small cell lung cancer/ or exp thyroid cancer/ or exp cancer cell line/ or exp "cancer of unknown primary site"/ or exp cancer prevention/ or exp breast cancer cell line/ or exp kidney cancer/ or exp bladder cancer/ or exp endometrium cancer/ or exp anus cancer/ or exp cancer immunotherapy/ or exp "HCC cell line (cervical cancer)"/ or exp bone marrow cancer/ or exp cancer research/ or exp metastatic breast cancer/ or exp urinary tract cancer/ or exp cancer inhibition/ or exp basal like breast cancer/ or exp cancer immunization/ or exp progesterone receptor positive breast cancer/
6	1 or 2 or 3 or 4 or 5
7	missing data.mp.
8	drop out.mp.
9	non-response.mp.
10	incomplete data.mp.
11	exclude* data.mp.
12	7 or 8 or 9 or 10 or 11

Table 1: Search Terms (continued)

#	Terms
13	6 and 12
14	exp survival analysis/
15	hazard ratio.mp.
16	time-to-event analysis.mp.
17	cox model.mp. or Proportional Hazards Models/
18	time-dependent.mp.
19	time varying.mp.
20	relative ratio.mp.
21	14 or 15 or 16 or 17 or 18 or 19 or 20
22	13 and 21
23	22 and 2012:2018.(sa_year).

2 Data extraction checklist

Table 2: Checklist for data extraction

Heading	Checklist	More detail
Journal		
Year		
First Author		
Analysis	Models used	• Kaplan-Meier<br>• Log rank test<br>• Cox model<br>• Exponential<br>• Weibull
	Complexity of analysis model	• Univariable<br>• Multivariable
	Functional form	• Checked?<br>• If yes, Martingale residuals?<br>• Other method?
	PH assumption	• Checked?<br>• Schoenfeld Residuals<br>• KM or log-log plots<br>• Interaction with time<br>• If other, specify<br>• If checked, was there an attempt to handle missing data other than a CC analysis?

PH= proportional hazards

Table 2: Checklist for data extraction (continued)

Heading	Checklist	More detail
Analysis	Covariate selection	• Explicitly stated?<br>• A priori involved?<br>• Univariable model with $p < \alpha$<br>• Chi-square<br>• T-test<br>• Fishers exact test<br>• Likelihood ratio test<br>• Forward selection<br>• Backward selection<br>• Other, specify
Missing data	Outside of CC analysis, was extent of missing specified	• in text<br>• in table<br>• shown in a plot
	Assumptions stated?	• If yes, which one?<br>• If no, presumably which one?
	Methods to handle missing data	• Were methods declared in full text or supplementary material?<br>• Initial size of sample (before excluding due to missing data or other criteria)<br>• After applying exclusion criteria not relating to missing data, was it unclear whether there were any missing covariate data?

Table 2: Checklist for data extraction (continued)

Heading	Checklist	More detail
Missing data	Methods to handle missing data	• Out of the initial sample size, how many subjects were excluded in an initial phase (prior to any descriptive statistics or analysis) because they had missing data on one or more covariates?<br>• Out of the initial sample size, how many subjects were excluded in an initial phase (prior to any descriptive statistics or analysis) due to other exclusion criteria?<br>• Did the reporting make it possible to ascertain the numbers excluded due to missing data or due to other exclusion criteria?<br>• If the reporting did not make it possible to ascertain the numbers excluded due to missing data or due to other exclusion criteria, what was the total number excluded in the initial phase for any reason?<br>• Of which, was it possible to determine at least some of the exclusion was due to missing data?<br>• If yes, how many were confirmed to be excluded due to missing data?<br>• Final sample size after all exclusion criteria applied to be used for analysis?

Table 2: Checklist for data extraction (continued)

Heading	Checklist	More detail
Missing data	Methods to handle missing data	• If excluded missing data, what would sample size for analysis have been if missing data had been kept?<br>• After exclusions in an initial phase due to either missing data and/or other criteria, are there missing values in any additional study covariates?<br>• If missing data still present in analysis sample, were covariates containing Nas used in model?<br>• Did initial phase include removing those with incomplete data in some covariates?<br>• Did initial phase include a CC analysis?<br>• Was complete-case analysis used during or post initial phase?<br>• Stated no. of people with complete records?<br>• If no, could no. of complete records be worked out from information in paper?<br>• Removed covariates from analysis due to large amount of missing data?<br>• If removed, are they thought to be highly predictive?

Table 2: Checklist for data extraction (continued)

Heading	Checklist	More detail
Missing data	Methods to handle missing data	• Included a missing indicator in model?<br>• Minimum value imputation<br>• Maximum value imputation<br>• Mean value imputation<br>• Mode value imputation<br>• LOCF<br>• Was MI used?<br>• If yes, uni or multi?<br>• Specify if Joint MVN, FCS etc.<br>• If yes, included time, log time, event indicator or Nelson-Aalen estimate?<br>• If yes, specify no. of imputations?<br>• Was a sensitivity analysis used?<br>• Were there any Time-dependent covariates or time-varying effects with missing data?
Software used		State whether SAS, SPSS, Stata, R, S-plus or Mplus

3 Papers included in the review

References

[1] Laura Bredow, Lisa Stutzel, Daniel Bohringer, Enken Gundlach, Thomas Reinhard, and Claudia Auw-Haedrich. Progesterone and estrogen receptors in conjunctival melanoma and nevi. *Graefe's archive for clinical and experimental ophthalmology = Albrecht von Graefes Archiv fur klinische und experimentelle Ophthalmologie*, 252(2):359–365, 2014.

[2] Benjamin Kasenda, Annatina Bass, Dieter Koeberle, Bernhard Pestalozzi, Markus Borner, Richard Herrmann, Lorenz Jost, Andreas Lohri, and Viviane Hess. Survival in overweight patients with advanced pancreatic carcinoma: a multicentre cohort study. *BMC cancer*, 14:728, 2014.

[3] Rajesh Sehgal, Mohamed Alsharedi, Chris Larck, Phyllis Edwards, and Todd Gress. Pancreatic cancer survival in elderly patients treated with chemotherapy. *Pancreas*, 43(2):306–310, 2014.

[4] Bradshaw P.T., Ibrahim J.G., Khankari N., Cleveland R.J., Abrahamson P.E., Stevens J., Satia J.A., Teitelbaum S.L., Neugut A.I., and Gammon M D. Post-diagnosis physical activity and survival after breast cancer diagnosis: The Long Island Breast Cancer Study. *Breast Cancer Research and Treatment*, 145(3):735–742, 2014.

[5] Daniel M Halperin, Chan Shen, Arvind Dasari, Ying Xu, Yiyi Chu, Shouhao Zhou, Ya-Chen Tina Shih, and James C Yao. Frequency of carcinoid syndrome at neuroendocrine tumour diagnosis: a population-based study. *The Lancet. Oncology*, 18(4):525–534, apr 2017.

[6] Milan Risteski, Simonida Crvenkova, Zoran Atanasov, and Rozalinda Isjanovska. Epidemiological analysis of progression-free survival (PFS) and overall survival (OS) in non-small-cell lung cancer patients in Republic of Macedonia. *Prilozi (Makedonska akademija na naukite i umetnostite. Oddelenie za medicinski nauki)*, 34(3):49–61, 2013.

[7] Annukka Pasanen, Taru Tuomi, Jorma Isola, Synnove Staff, Ralf Butzow, and Mikko Loukovaara. L1 Cell Adhesion Molecule as a Predictor of Disease-Specific Survival and Patterns of Relapse in Endometrial Cancer. *International journal of gynecological cancer : official journal of the International Gynecological Cancer Society*, 26(8):1465–1471, 2016.

[8] Andrew J Kaufman, Justin Palatt, Mark Sivak, Peter Raimondi, Dong-Seok Lee, Andrea Wolf, Fouad Lajam, Faiz Bhora, and Raja M Flores. Thymectomy for

Myasthenia Gravis: Complete Stable Remission and Associated Prognostic Factors in Over 1000 Cases. *Seminars in thoracic and cardiovascular surgery*, 28(2):561–568, 2016.

[9] Patrick T Bradshaw, Joseph G Ibrahim, June Stevens, Rebecca Cleveland, Page E Abrahamson, Jessie A Satia, Susan L Teitelbaum, Alfred I Neugut, and Marilie D Gammon. Postdiagnosis change in bodyweight and survival after breast cancer diagnosis. *Epidemiology*, 23(2):320–327, 2012.

[10] Makatsoris T., Tsamandas A.C., Strimpakos A., Alexopoulou Z., Dionysopoulos D., Pervana S., Konstantara A., Papakostas P., Samantas E., Rallis G., Dimou A., Pentheroudakis G., Papaparaskeva K., Psyrri A., Kalogeras K.T., Syrigos K., and Scopa C.D. HER family protein expression in a Greek population with gastric cancer. A retrospective hellenic cooperative oncology group study. *Anticancer Research*, 36(4):1581–1590, 2016.

[11] Keto C.J., Aronson W.J., Terris M.K., Presti J.C., Kane C.J., and Amling C.L. Obesity is associated with castration-resistant disease and metastasis in men treated with androgen deprivation therapy after radical prostatectomy: Results from the SEARCH database. *BJU International*, 110(4):492–498, 2012.

[12] Lubna Alhalabi, Matthew J Singleton, Abdullahi O Oseni, Amit J Shah, Zhu-Ming Zhang, and Elsayed Z Soliman. Relation of Higher Resting Heart Rate to Risk of Cardiovascular Versus Noncardiovascular Death. *The American journal of cardiology*, 119(7):1003–1007, apr 2017.

[13] R Elaidi, A Harbaoui, B Beuselinck, J-C Eymard, A Bamias, E De Guillebon, C Porta, Y Vano, C Linassier, P R Debruyne, M Gross-Goupil, A Ravaud, M Aitelhaj, G Marret, and S Oudard. Outcomes from second-line therapy in long-term responders to first-line tyrosine kinase inhibitor in clear-cell metastatic renal cell carcinoma. *Annals of oncology : official journal of the European Society for Medical Oncology*, 26(2):378–385, 2015.

[14] Amini A., Rusthoven C.G., Jones B.L., Armstrong H., and Raben D. Survival outcomes of radiotherapy with or without androgen-deprivation therapy for patients with intermediate-risk prostate cancer using the National Cancer Data Base. *Urologic Oncology: Seminars and Original Investigations*, 34(4):165, 2016.

[15] Joseph C. Y. Chan, Connie I. Diakos, David L. H. Chan, Alexander Engel, Nick Pavlakis, Anthony Gill, and Stephen J. Clarke. A Longitudinal Investigation of Inflammatory Markers in Colorectal Cancer Patients Perioperatively Demonstrates Benefit in Serial Remeasurement. *Annals of Surgery*, page 1, apr 2017.

[16] Michael E Egger, Brittany L Tabler, Erik M Dunki-Jacobs, Glenda G Callender, Charles R Scoggins, Robert C G 2nd Martin, Amy R Quillo, Arnold J Stromberg, and Kelly M McMasters. Clinicopathologic and survival differences between upper and lower extremity melanomas. *The American surgeon*, 78(7):779–787, 2012.

[17] Alper Biler, Ulas Solmaz, Selcuk Erkilinc, Mehmet Gokcu, Mustafa Bagci, Orhan Temel, Tugba Karadeniz, and Muzaffer Sanci. Analysis of endometrial carcinoma in young women at a high-volume cancer center. *International Journal of Surgery*, 44:185–190, aug 2017.

[18] Marko Lukic, Idlir Licaj, Eiliv Lund, Guri Skeie, Elisabete Weiderpass, and Tonje Braaten. Coffee consumption and the risk of cancer in the Norwegian Women and Cancer (NOWAC) Study. *European Journal of Epidemiology*, 31(9):905–916, sep 2016.

[19] Andrea Rocca, Alberto Farolfi, Roberta Maltoni, Elisa Carretta, Elisabetta Melegari, Cristiano Ferrario, Lorenzo Cecconetto, Samanta Sarti, Alessio Schirone, Anna Fedeli, Daniele Andreis, Elisabetta Pietri, Toni Ibrahim, Erika Montalto, and Dino Amadori. Efficacy of endocrine therapy in relation to progesterone receptor and Ki67 expression in advanced breast cancer. *Breast cancer research and treatment*, 152(1):57–65, 2015.

[20] Eggemann H., Ignatov T., Burger E., Kantelhardt E.J., Fettke F., Thomssen C., and Dan Costa S. Moderate HER2 expression as a prognostic factor in hormone receptor positive breast cancer. *Endocrine-Related Cancer*, 22(5):725–733, 2015.

[21] Yong Jin Kang, Won Sik Jang, Jong Kyou Kwon, Cheol Yong Yoon, Joo Yong Lee, Won Sik Ham, and Young Deuk Choi. Intermediate PSA half-life after neoadjuvant hormone therapy predicts reduced risk of castration-resistant prostate cancer development after radical prostatectomy. *BMC Cancer*, 17(1):789, dec 2017.

[22] Rafael Bitzur, Ronen Brenner, Elad Maor, Maayan Antebi, Tomer Ziv-Baran, Shlomo Segev, Yechezkel Sidi, and Shaye Kivity. Metabolic syndrome, obesity, and the risk of cancer development. *European journal of internal medicine*, 34:89–93, 2016.

[23] Oladeru O.T., Miccio J.A., Yang J., Xue Y., and Ryu S. Conformal external beam radiation or selective internal radiation therapy-a comparison of treatment outcomes for hepatocellular carcinoma. *Journal of Gastrointestinal Oncology*, 7(3):433–440, 2016.

[24] Sarah Krull Abe, Manami Inoue, Norie Sawada, Junko Ishihara, Motoki Iwasaki, Taiki Yamaji, Taichi Shimazu, Shizuka Sasazuki, and Shoichiro Tsugane. Glycemic

index and glycemic load and risk of colorectal cancer: a population-based cohort study (JPHC Study). *Cancer causes & control : CCC*, 27(4):583–593, 2016.

[25] Alfonso Rivera Duarte, Alejandra Armengol Alonso, Elena Sandoval Cartagena, and Elena Tuna Aguilar. Blastic Transformation in Mexican Population With Chronic Myelomonocytic Leukemia. *Clinical Lymphoma Myeloma and Leukemia*, 17(8):532–538, aug 2017.

[26] Sun G.E.C., Wells B.J., Yip K., Zimmerman R., Raghavan D., and Kattan M.W. Gender-specific effects of oral hypoglycaemic agents on cancer risk in type 2 diabetes mellitus. *Diabetes, Obesity and Metabolism*, 16(3):276–283, 2014.

[27] Lindsay A Renfro, Axel Grothey, Yuan Xue, Leonard B Saltz, Thierry Andre, Chris Twelves, Roberto Labianca, Carmen J Allegra, Steven R Alberts, Charles L Loprinzi, Greg Yothers, Daniel J Sargent, and Adjuvant Colon Cancer Endpoints (ACCENT) Group. ACCENT-based web calculators to predict recurrence and overall survival in stage III colon cancer. *Journal of the National Cancer Institute*, 106(12), 2014.

[28] Dante Wan, Diego Villa, Ryan Woods, Rinat Yerushalmi, and Karen Gelmon. Breast Cancer Subtype Variation by Race and Ethnicity in a Diverse Population in British Columbia. *Clinical breast cancer*, 16(3):e49–55, 2016.

[29] Shamseddine A.I., Mukherji D., Melki C., Elias E., Eloubeidi M., Dimassi H., Khalife M., Abou-Alfa G., and O'Reilly E. Lymph node ratio is an independent prognostic factor after resection of periampullary malignancies: Data from a tertiary referral center in the middle east. *American Journal of Clinical Oncology: Cancer Clinical Trials*, 37(1):13–18, 2014.

[30] Strimpakos A., Pentheroudakis G., Kotoula V., De Roock W., Kouvatseas G., Papakostas P., Makatsoris T., Papamichael D., Andreadou A., Sgouros J., Zizi-Sermpetzoglou A., Kominea A., Televantou D., Razis E., Galani E., Pectasides D., Tejpar S., and Syrigos K. The prognostic role of ephrin A2 and endothelial growth factor receptor pathway mediators in patients with advanced colorectal cancer treated with cetuximab. *Clinical Colorectal Cancer*, 12(4):267, 2013.

[31] Vivek Thumbigere-Math, Lam Tu, Sabrina Huckabay, Arkadiusz Z Dudek, Scott Lunos, David L Basi, Pamela J Hughes, Joseph W Leach, Karen K Swenson, Rajaram Gopalakrishnan, Thumbigere-Math V., Tu L., Huckabay S., Dudek A.Z., Lunos S., Basi D.L., Hughes P.J., Leach J.W., and Swenson K.K. A retrospective study evaluating frequency and risk factors of osteonecrosis of the jaw in 576 cancer patients receiving intravenous bisphosphonates. *American journal of clinical oncology*, 35(4):386–392, 2012.

[32] Sjoblom B., Gronberg B.H., Wentzel-Larsen T., Baracos V.E., Hjermstad M.J., Aass N., Bremnes R.M., Flotten O., and Bye A. Skeletal muscle radiodensity is prognostic for survival in patients with advanced non-small cell lung cancer. *Clinical Nutrition*, 35(6):1386–1393, 2016.

[33] Kyle A Richards, Joshua A Cohn, Michael C Large, Gregory T Bales, Norm D Smith, and Gary D Steinberg. The effect of length of ureteral resection on benign ureterointestinal stricture rate in ileal conduit or ileal neobladder urinary diversion following radical cystectomy. *Urologic oncology*, 33(2):65.e1–8, 2015.

[34] Daniel A Morgenstern, Wendy B London, Derek Stephens, Samuel L Volchenboum, Barbara Hero, Andrea Di Cataldo, Akira Nakagawara, Hiroyuki Shimada, Peter F Ambros, Katherine K Matthay, Susan L Cohn, Andrew D J Pearson, and Meredith S Irwin. Metastatic neuroblastoma confined to distant lymph nodes (stage 4N) predicts outcome in patients with stage 4 disease: A study from the International Neuroblastoma Risk Group Database. *Journal of clinical oncology : official journal of the American Society of Clinical Oncology*, 32(12):1228–1235, 2014.

[35] Michael A Liss, Martha White, Loki Natarajan, and J Kellogg Parsons. Exercise Decreases and Smoking Increases Bladder Cancer Mortality. *Clinical genitourinary cancer*, 15(3):391–395, jun 2017.

[36] Laura J Rasmussen-Torvik, Christina M Shay, Judith G Abramson, Christopher A Friedrich, Jennifer A Nettleton, Anna E Prizment, and Aaron R Folsom. Ideal cardiovascular health is inversely associated with incident cancer: the Atherosclerosis Risk In Communities study. *Circulation*, 127(12):1270–1275, 2013.

[37] Katharine Bailey, Andy Ryan, Sophia Apostolidou, Evangelia Fourkala, Matthew Burnell, Aleksandra Gentry-Maharaj, Jatinderpal Kalsi, Max Parmar, Ian Jacobs, Hynek Pikhart, and Usha Menon. Socioeconomic indicators of health inequalities and female mortality: a nested cohort study within the United Kingdom Collaborative Trial of Ovarian Cancer Screening (UKCTOCS). *BMC public health*, 15:253, 2015.

[38] Min J.-Y. Blood trihalomethane levels and the risk of total cancer mortality in US adults. *Environmental Pollution*, 212:90–96, 2016.

[39] A M Behie and M H O'Donnell. Prenatal smoking and age at menarche: influence of the prenatal environment on the timing of puberty. *Human reproduction (Oxford, England)*, 30(4):957–962, 2015.

[40] Mehta R., Gillan A.S., Ming Z.Y., Rai B.P., and Byrne D. Socio-economic deprivation and outcomes following radical nephroureterectomy for clinically localized upper tract transitional cell carcinoma. *World journal of urology*, 33(1):41–49, 2015.

[41] Ohri N., Duan F., MacHtay M., Gorelick J.J., Snyder B.S., Alavi A., Siegel B.A., Johnson D.W., Bradley J.D., and Denittis A. Pretreatment FDG-PET metrics in stage III non-small cell lung cancer: ACRIN 6668/RTOG 0235. *Journal of the National Cancer Institute*, 107(4), 2015.

[42] Lino-Silva L.S., Dominguez-Rodriguez J.A., Aguilar-Romero J.M., Martinez-Said H., Salcedo-Hernandez R.A., Garcia-Perez L., and Herrera-Gomez A. Melanoma in Mexico: Clinicopathologic Features in a Population with Predominance of Acral Lentiginous Subtype. *Annals of Surgical Oncology*, 23(13):4189–4194, 2016.

[43] Anna Vogiatzoglou, Angela A Mulligan, Amit Bhaniani, Marleen A H Lentjes, Alison McTaggart, Robert N Luben, Christian Heiss, Malte Kelm, Marc W Merx, Jeremy P E Spencer, Hagen Schroeter, Kay-Tee Khaw, and Gunter G C Kuhnle. Associations between flavan-3-ol intake and CVD risk in the Norfolk cohort of the European Prospective Investigation into Cancer (EPIC-Norfolk). *Free radical biology & medicine*, 84:1–10, 2015.

[44] Kelly M Cordoro, Deepti Gupta, Ilona J Frieden, Timothy McCalmont, and Mohammed Kashani-Sabet. Pediatric melanoma: results of a large cohort study and proposal for modified ABCD detection criteria for children. *Journal of the American Academy of Dermatology*, 68(6):913–925, 2013.

[45] Zaragoza J., Kervarrec T., Touze A., Avenel-Audran M., Beneton N., Esteve E., Wierzbicka Hainaut E., Aubin F., Machet L., Julia Zaragoza, Thibault Kervarrec, Antoine Touze, Martine Avenel-Audran, Nathalie Beneton, Eric Esteve, Ewa Wierzbicka Hainaut, Francois Aubin, Laurent Machet, and Mahtab Samimi. A high neutrophil-to-lymphocyte ratio as a potential marker of mortality in patients with Merkel cell carcinoma: A retrospective study. *Journal of the American Academy of Dermatology*, 75(4):712–721.e1, 2016.

[46] Marit Busund, Nora S. Bugge, Tonje Braaten, Marit Waaseth, Charlotta Rylander, and Eiliv Lund. Progestin-only and combined oral contraceptives and receptor-defined premenopausal breast cancer risk: The Norwegian Women and Cancer Study. *International Journal of Cancer*, feb 2018.

[47] Lin-Hui Su, Li-Sheng Chen, Sheng-Che Lin, and Hsiu-Hsi Chen. Association of androgenetic alopecia with mortality from diabetes mellitus and heart disease. *JAMA dermatology*, 149(5):601–606, 2013.

[48] N Saade, C Sadler, and M Goldfarb. Impact of Regional Lymph Node Dissection on Disease Specific Survival in Adrenal Cortical Carcinoma. *Hormone and metabolic research = Hormon- und Stoffwechselforschung = Hormones et metabolisme*, 47(11):820–825, 2015.

[49] Daniel Orbach, Bernadette Brennan, Gianni Bisogno, Max Van Noesel, Véronique Minard-Colin, Julia Daragjati, Michela Casanova, Nadege Corradini, Ilaria Zanetti, Gian Luca De Salvo, Anne Sophie Defachelles, Anna Kelsey, Myriam Ben Arush, Nadine Francotte, and Andrea Ferrari. The EpSSG NRSTS 2005 treatment protocol for desmoid-type fibromatosis in children: an international prospective case series. *The Lancet Child & Adolescent Health*, 1(4):284–292, dec 2017.

[50] Zeljka Jutric, Jan Grendar, Helena M. Hoen, Sung W. Cho, Maria A. Cassera, Pippa H. Newell, Chet W. Hammill, Paul D. Hansen, and Ronald F. Wolf. Regional Metastatic Behavior of Nonfunctional Pancreatic Neuroendocrine Tumors. *Pancreas*, 46(7):898–903, aug 2017.

[51] Harding J.L., Shaw J.E., Anstey K.J., Adams R., Balkau B., Brennan-Olsen S.L., Briffa T., Davis T.M., Davis W.A., Dobson A., Flicker L., Giles G., Grant J., Huxley R., Knuiman M., Luszcz M., Macinnis R.J., Mitchell P., Pasco J.A., Reid C., Simmons D., Simons L., Tonkin A., Woodward M., and Peeters A. Comparison of anthropometric measures as predictors of cancer incidence: A pooled collaborative analysis of 11 Australian cohorts. *International Journal of Cancer*, 2015.

[52] Christopher J Keto, William J Aronson, Martha K Terris, Joseph C Presti, Christopher J Kane, Christopher L Amling, and Stephen J Freedland. Detectable prostate-specific antigen Nadir during androgen-deprivation therapy predicts adverse prostate cancer-specific outcomes: results from the SEARCH database. *European urology*, 65(3):620–627, 2014.

[53] Fornaro L., Cereda S., Aprile G., Di Girolamo S., Santini D., Silvestris N., Lonardi S., Leone F., Milella M., Vivaldi C., Belli C., Bergamo F., Lutrino S.E., Filippi R., Russano M., Vaccaro V., Brunetti A.E., Rotella V., Falcone A., Barbera M.A., Corbelli J., Fasola G., Aglietta M., Zagonel V., Reni M., and Vasile E. Multivariate prognostic factors analysis for second-line chemotherapy in advanced biliary tract cancer. *British Journal of Cancer*, 110(9):2165–2169, 2014.

[54] Hai-Xia Liu, Na Li, Li Wei, Fu-Xing Zhou, Rui Ma, Feng Xiao, Wei Zhang, Ying Zhang, Yan-Ping Hui, Hui Song, and Bi-Liang Chen. High expression of Kruppel-like factor 4 as a predictor of poor prognosis for cervical cancer patient response to radiotherapy. *Tumour biology : the journal of the International Society for Oncodevelopmental Biology and Medicine*, 39(6):1010428317710225, 2017.

[55] Rapat Pittayanon, Rungsun Rerknimitr, and Alan Barkun. Prognostic factors affecting outcomes in patients with malignant GI bleeding treated with a novel endoscopically delivered hemostatic powder. *Gastrointestinal Endoscopy*, 87(4):994–1002, apr 2018.

[56] Ahmed Elshafei, Kae Jack Tay, Onder Kara, Ercan Malkoc, Yaw Nyame, Hans Arora, Asmaa Hatem, Sahil A. Patel, Franco Lugnani, Thomas J. Polascik, and J. Stephen Jones. Associations Between Prostate Volume and Oncologic Outcomes in Men Undergoing Focal Cryoablation of the Prostate. *Clinical Genitourinary Cancer*, 16(2):e477–e482, apr 2018.

[57] Akbar Fazel-Tabar Malekshah, Marsa Zaroudi, Arash Etemadi, Farhad Islami, Sadaf Sepanlou, Maryam Sharafkhah, Abbas-Ali Keshtkar, Hooman Khademi, Hossein Poustchi, Azita Hekmatdoost, Akram Pourshams, Akbar Feiz Sani, Elham Jafari, Farin Kamangar, Sanford M Dawsey, Christian C Abnet, Paul D Pharoah, Paul J Berennan, Paolo Boffetta, Ahmad Esmaillzadeh, and Reza Malekzadeh. The Combined Effects of Healthy Lifestyle Behaviors on All-Cause Mortality: The Golestan Cohort Study. *Archives of Iranian medicine*, 19(11):752–761, 2016.

[58] Cheng Yuan, Ning Li, Xiaoyong Mao, Zui Liu, Wei Ou, and Si-Yu Wang. Elevated pretreatment neutrophil/white blood cell ratio and monocyte/lymphocyte ratio predict poor survival in patients with curatively resected non-small cell lung cancer: Results from a large cohort. *Thoracic cancer*, 8(4):350–358, jul 2017.

[59] Valerie A Smith, Roy B Sessions, and Eric J Lentsch. Cervical lymph node metastasis and papillary thyroid carcinoma: does the compartment involved affect survival? Experience from the SEER database. *Journal of surgical oncology*, 106(4):357–362, 2012.

[60] Sarah Kawaguchi Jaimin R. Bhatt, Michael A. S. Jewett, Patrick O. Richard and Antonio Finelli Narhari Timilshina, Andrew Evans, Shabbir Alibhai. Multilocular cystic renal cell carcinoma: pathological t staging makes no difference to favorable outcomes and should be reclassified. *The Journal of urology*, 196:1350–1355, 2016.

[61] A Necchi, R Miceli, M Bregni, C Bokemeyer, L A Berger, K Oechsle, K Schumacher, E Kanfer, J H Bourhis, C Massard, D Laszlo, J Montoro, A Flechon, F Arpaci, S Secondino, P Wuchter, P Dreger, M Crysandt, N Worel, W Kruger, M Ringhoffer, A Unal, A Nagler, A Campos, A Wahlin, M Michieli, G Sucak, I Donnini, R Schots, N Ifrah, M Badoglio, M Martino, D Raggi, P Giannatempo, G Rosti, P Pedrazzoli, and F Lanza. Prognostic impact of progression to induction chemotherapy and prior paclitaxel therapy in patients with germ cell tumors receiving salvage high-dose chemotherapy in the last 10 years: a study of the European Society for Blood and Marrow Transplantation S. *Bone marrow transplantation*, 51(3):384–390, 2016.

[62] E Susan Amirian, Terri S Armstrong, Kenneth D Aldape, Mark R Gilbert, and Michael E Scheurer. Predictors of survival among pediatric and adult ependymoma cases: a study using Surveillance, Epidemiology, and End Results data from 1973 to 2007. *Neuroepidemiology*, 39(2):116–124, 2012.

[63] McCabe E.L., Larson M.G., Lunetta K.L., Newman A.B., and Cheng S. Association of an Index of Healthy Aging With Incident Cardiovascular Disease and Mortality in a Community-Based Sample of Older Adults. *The journals of gerontology. Series A, Biological sciences and medical sciences*, 71(12):1695–1701, 2016.

[64] Alicja Puszkiel, Melanie White-Koning, Nicolas Dupin, Nora Kramkimel, Audrey Thomas-Schoemann, Gaelle Noe, Nicolas Chapuis, Michel Vidal, Francois Goldwasser, Etienne Chatelut, and Benoit Blanchet. Plasma vemurafenib exposure and pre-treatment hepatocyte growth factor level are two factors contributing to the early peripheral lymphocytes depletion in BRAF-mutated melanoma patients. *Pharmacological research*, 113(Pt A):709–718, 2016.

[65] Piet A van den Brandt and Maya Schulpen. Mediterranean diet adherence and risk of postmenopausal breast cancer: results of a cohort study and meta-analysis. *International journal of cancer*, 140(10):2220–2231, may 2017.

[66] Ferguson M.K., Watson S., and Johnson E. Predicted postoperative lung function is associated with all-cause long-term mortality after major lung resection for cancer. *European Journal of Cardio-thoracic Surgery*, 45(4):660–664, 2014.

[67] M Yi, L Huo, K B Koenig, E A Mittendorf, F Meric-Bernstam, H M Kuerer, I Bedrosian, A U Buzdar, W F Symmans, J R Crow, M Bender, R R Shah, G N Hortobagyi, and K K Hunt. Which threshold for ER positivity? a retrospective study based on 9639 patients. *Annals of oncology : official journal of the European Society for Medical Oncology*, 25(5):1004–1011, 2014.

[68] Lv J.-W., Chen Y.-P., Zhou G.-Q., Tang L.-L., Mao Y.-P., Li W.-F., Guo R., Lin A.-H., and Ma J. Cigarette smoking complements the prognostic value of baseline plasma Epstein-Barr virus deoxyribonucleic acid in patients with nasopharyngeal carcinoma undergoing intensity-modulated radiation therapy: A large-scale retrospective cohort study. *Oncotarget*, 7(13):16806–16817, 2016.

[69] John J Coen, Jonathan J Paly, Andrzej Niemierko, Donald S Kaufman, Niall M Heney, Daphne Y Spiegel, Jason A Efstathiou, Anthony L Zietman, and William U Shipley. Nomograms predicting response to therapy and outcomes after bladder-preserving trimodality therapy for muscle-invasive bladder cancer. *International journal of radiation oncology, biology, physics*, 86(2):311–316, 2013.

[70] Takahashi M., Komine K., Yamada H., Kasahara Y., Chikamatsu S., Okita A., Ito S., Ouchi K., Okada Y., Imai H., Saijo K., Shimodaira Shirota H., Takahashi S., Mori T., Shimodaira Shirota H., Masahiro Masanobu Takahashi, Masahiro Masanobu Takahashi, Keigo Komine, Hideharu Yamada, Yuki Kasahara, Sonoko Chikamatsu, Akira Okita, Shukuei Ito, Kota Ouchi, Yoshinari Okada, Hiroo Imai, Ken Saijo,

Hidekazu Shirota, Shin Takahashi, Takahiro Mori, Hideki Shimodaira, and Chikashi Ishioka. The G8 screening tool enhances prognostic value to ECOG performance status in elderly cancer patients: A retrospective, single institutional study. *PLoS ONE*, 12(6):e0179694, 2017.

[71] Jens Kohler, Martin Schuler, Thomas Christoph Gauler, Stefanie Nopel-Dunnebacke, Maike Ahrens, Andreas-Claudius Hoffmann, Stefan Kasper, Felix Nensa, Benedikt Gomez, Maria Hahnemann, Frank Breitenbuecher, Danjouma Cheufou, Filiz Ozkan, Kaid Darwiche, Mathias Hoiczyk, Henning Reis, Stefan Welter, Wilfried Ernst Erich Eberhardt, Martin Eisenacher, Helmut Teschler, Georgios Stamatis, Wolff Schmiegel, Stephan Albrecht Hahn, and Alexander Baraniskin. Circulating U2 small nuclear RNA fragments as a diagnostic and prognostic biomarker in lung cancer patients. *Journal of cancer research and clinical oncology*, 142(4):795–805, 2016.

[72] Paly J.J., Hallemeier C.L., Biggs P.J., Niemierko A., Roeder F., Martinez-Monge R., Whitson J., Calvo F.A., Fastner G., Sedlmayer F., Wong W.W., Ellis R.J., Haddock M.G., Choo R., Shipley W.U., and Zietman A.L. Outcomes in a multi-institutional cohort of patients treated with intraoperative radiation therapy for advanced or recurrent renal cell carcinoma. *International Journal of Radiation Oncology Biology Physics*, 88(3):618–623, 2014.

[73] Gabriel E., Attwood K., Thirunavukarasu P., Al-Sukhni E., Boland P., Emmanuel Gabriel, Kristopher Attwood, Pragatheeshwar Thirunavukarasu, Eisar Al-Sukhni, Patrick Boland, and Steven Nurkin. Predicting Individualized Postoperative Survival for Stage II/III Colon Cancer Using a Mobile Application Derived from the National Cancer Data Base. *Journal of the American College of Surgeons*, 222(3):232–244, 2016.

[74] Marko Lukic, Mie Jareid, Elisabete Weiderpass, and Tonje Braaten. Coffee consumption and the risk of malignant melanoma in the Norwegian Women and Cancer (NOWAC) Study. *BMC cancer*, 16:562, 2016.

[75] G Gandaglia, G Lista, N Fossati, N Suardi, A Gallina, M Moschini, L Bianchi, M S Rossi, R Schiavina, S F Shariat, A Salonia, F Montorsi, and A Briganti. Non-surgically related causes of erectile dysfunction after bilateral nerve-sparing radical prostatectomy. *Prostate cancer and prostatic diseases*, 19(2):185–190, 2016.

[76] Guru Sonpavde, Gregory R Pond, Andrew J Armstrong, Stephen J Clarke, Janette L Vardy, Arnoud J Templeton, Shaw-Ling Wang, Jolanda Paolini, Isan Chen, Edna Chow-Maneval, Mariajose Lechuga, Matthew R Smith, and M Dror Michaelson. Prognostic impact of the neutrophil-to-lymphocyte ratio in men with metastatic

castration-resistant prostate cancer. *Clinical genitourinary cancer*, 12(5):317–324, 2014.

[77] Geoffrey C Kabat, Charles E Matthews, Victor Kamensky, Albert R Hollenbeck, and Thomas E Rohan. Adherence to cancer prevention guidelines and cancer incidence, cancer mortality, and total mortality: a prospective cohort study. *The American journal of clinical nutrition*, 101(3):558–569, 2015.

[78] Li J., Eriksson M., Czene K., and Hall P. Common diseases as determinants of menopausal age. *Human Reproduction*, 31(12):2856–2864, 2016.

[79] B E Shaw, N P Mayor, R M Szydlo, W P Bultitude, C Anthias, K Kirkland, J Perry, A Clark, S Mackinnon, D I Marks, A Pagliuca, M N Potter, N H Russell, K Thomson, J A Madrigal, and S G E Marsh. Recipient/donor HLA and CMV matching in recipients of T-cell-depleted unrelated donor haematopoietic cell transplants. *Bone marrow transplantation*, 52(5):717–725, 2017.

[80] Melina Arnold, Luohua Jiang, Marcia L Stefanick, Karen C Johnson, Dorothy S Lane, Erin S LeBlanc, Ross Prentice, Thomas E Rohan, Beverly M Snively, Mara Vitolins, Oleg Zaslavsky, Isabelle Soerjomataram, Hoda Anton-Culver, Arnold M., Jiang L., Stefanick M.L., Johnson K.C., Lane D.S., LeBlanc E.S., Prentice R., Rohan T.E., Snively B.M., Vitolins M., Zaslavsky O., Soerjomataram I., and Melina; ORCID: http://orcid.org/0000-0003-1700-6831 A O Jiang Anton-Culver H. AO - Arnold Luohua; ORCID: http://orcid.org/0000-0002-2281-7260. Duration of Adulthood Overweight, Obesity, and Cancer Risk in the Women's Health Initiative: A Longitudinal Study from the United States. *PLoS medicine*, 13(8):e1002081, 2016.

[81] Atsumu Yuki, Rei Otsuka, Chikako Tange, Yukiko Nishita, Makiko Tomida, Fujiko Ando, and Hiroshi Shimokata. Physical frailty and mortality risk in Japanese older adults. *Geriatrics & Gerontology International*, apr 2018.

[82] Brandon-Luke L. Seagle, Amy L. Alexander, Taliya Lantsman, and Shohreh Shahabi. Prognosis and treatment of positive peritoneal cytology in early endometrial cancer: matched cohort analyses from the National Cancer Database. *American Journal of Obstetrics and Gynecology*, 218(3):329.e1–329.e15, mar 2018.

[83] Elizabeth Kersten, Patricia Scanlan, Steven G Dubois, and Katherine K Matthay. Current treatment and outcome for childhood acute leukemia in Tanzania. *Pediatric blood & cancer*, 60(12):2047–2053, 2013.

[84] Masahiro Yanagiya, Jun-ichi Nitadori, Kazuhiro Nagayama, Masaki Anraku, Masaaki Sato, and Jun Nakajima. Prognostic significance of the preoperative neutrophil-to-lymphocyte ratio for complete resection of thymoma. *Surgery Today*, 48(4):422–430, apr 2018.

[85] Miller R.E., Markt S.C., O'Donnell E., Bernard B., Albiges L.K., and Beard C. Age >=40 Years Is Associated with Adverse Outcome in Metastatic Germ Cell Cancer Despite Appropriate Intended Chemotherapy. *European Urology Focus*, 2016.

[86] Sara Hallden, Marketa Sjogren, Bo Hedblad, Gunnar Engstrom, Krzysztof Narkiewicz, Michal Hoffmann, Bjorn Wahlstrand, Thomas Hedner, and Olle Melander. Smoking and obesity associated BDNF gene variance predicts total and cardiovascular mortality in smokers. *Heart (British Cardiac Society)*, 99(13):949–953, 2013.

[87] Katherine A Janeway, Donald A Barkauskas, Mark D Krailo, Paul A Meyers, Cindy L Schwartz, David H Ebb, Nita L Seibel, Holcombe E Grier, Richard Gorlick, and Neyssa Marina. Outcome for adolescent and young adult patients with osteosarcoma: a report from the Children's Oncology Group. *Cancer*, 118(18):4597–4605, 2012.

[88] David B Stewart, Christopher Hollenbeak, Susan Desharnais, Fabian Camacho, Patricia Gladowski, Vickie L Goff, and Li Wang. Rectal cancer and teaching hospitals: hospital teaching status affects use of neoadjuvant radiation and survival for rectal cancer patients. *Annals of surgical oncology*, 20(4):1156–1163, 2013.

[89] Maria Iachina, Erik Jakobsen, Anne Kudsk Fallesen, and Anders Green. Transfer between hospitals as a predictor of delay in diagnosis and treatment of patients with Non-Small Cell Lung Cancer - a register based cohort-study. *BMC health services research*, 17(1):267, 2017.

[90] Wu S.-G., Zhang Z.-Q., Liu W.-M., He Z.-Y., Li F.-Y., Lin H.-X., Sun J.-Y., and Lin H. Impact of the number of resected lymph nodes on survival after preoperative radiotherapy for esophageal cancer. *Oncotarget*, 7(16):22497–22507, 2016.

[91] Di Lorenzo G., Buonerba C., Bellelli T., Romano C., Montanaro V., Ferro M., Benincasa A., Ribera D., Lucarelli G., De Cobelli O., Sonpavde G., Giuseppe Di Lorenzo, Carlo Buonerba, Teresa Bellelli, Concetta Romano, Vittorino Montanaro, Matteo Ferro, Alfonso Benincasa, Dario Ribera, Giuseppe Lucarelli, Ottavio De Cobelli, Guru Sonpavde, Sabino De Placido, Di Lorenzo G., Buonerba C., Bellelli T., Romano C., Montanaro V., Ferro M., Benincasa A., Ribera D., Lucarelli G., De Cobelli O., Sonpavde G., and De Placido S. Third-Line chemotherapy for metastatic Urothelial Cancer: A retrospective observational study. *Medicine*, 94(51):e2297, 2015.

[92] Amanda I Phipps, Paul J Limburg, John A Baron, Andrea N Burnett-Hartman, Daniel J Weisenberger, Peter W Laird, Frank A Sinicrope, Christophe Rosty, Daniel D Buchanan, John D Potter, and Polly A Newcomb. Association between

molecular subtypes of colorectal cancer and patient survival. *Gastroenterology*, 148(1):77–87.e2, 2015.

[93] Marco Carbone, Alessandra Nardi, Tania Marianelli, Kate Martin, Alex Hudson, David Collett, Renato Romagnoli, Antonio Pinna, Alexander Gimson, James M Neuberger, Mario Angelico, , Liver Match Investigators for Italian Association for the Study of the Liver National Transplant Centre Transplant, and the National Health System Blood. International comparison of liver transplant programmes: differences in indications, donor and recipient selection and outcome between Italy and UK. *Liver international : official journal of the International Association for the Study of the Liver*, 36(10):1481–1489, 2016.

[94] Thompson E.M., Hielscher T., Bouffet E., Remke M., Luu B., Gururangan S., McLendon R.E., Bigner D.D., Lipp E.S., Perreault S., Cho Y.-J., Grant G., Kim S.-K., Lee J.Y., Rao A.A.N., Giannini C., Li K.K.W., Ng H.-K., Yao Y., Kumabe T., Tominaga T., Grajkowska W.A., Perek-Polnik M., Low D.C.Y., Seow W.T., Chang K.T.E., Mora J., Pollack I.F., Hamilton R.L., Leary S., Moore A.S., Ingram W.J., Hallahan A.R., Jouvet A., Fevre-Montange M., Vasiljevic A., Faure-Conter C., Shofuda T., Kagawa N., Hashimoto N., Jabado N., Weil A.G., Gayden T., Wataya T., Shalaby T., Grotzer M., Zitterbart K., Sterba J., Kren L., Hortobagyi T., Klekner A., Laszlo B., Pocza T., Hauser P., Schuller U., Jung S., Jang W.-Y., French P.J., Kros J.M., van Veelen M.-L.C., Massimi L., Leonard J.R., Rubin J.B., Vibhakar R., Chambless L.B., Cooper M.K., Thompson R.C., Faria C.C., Carvalho A., Nunes S., Pimentel J., Fan X., Muraszko K.M., Lopez-Aguilar E., Lyden D., Garzia L., Shih D.J.H., Kijima N., Schneider C., Adamski J., Northcott P.A., Kool M., Jones D.T.W., Chan J.A., Nikolic A., Garre M.L., Van Meir E.G., Osuka S., Olson J.J., Jahangiri A., Castro B.A., Gupta N., Weiss W.A., Moxon-Emre I., Mabbott D.J., Lassaletta A., Hawkins C.E., Tabori U., Drake J., Kulkarni A., Dirks P., Rutka J.T., Korshunov A., Pfister S.M., Packer R.J., and Ramaswamy V. Prognostic value of medulloblastoma extent of resection after accounting for molecular subgroup: a retrospective integrated clinical and molecular analysis. *The Lancet Oncology*, 17(4):484–495, 2016.

[95] Farkhad Manapov, Maximilian Niyazi, Sabine Gerum, Olarn Roengvoraphoj, Chukwuka Eze, Minglun Li, Guido Hildebrandt, Rainer Fietkau, Gunther Klautke, and Claus Belka. Evaluation of the role of remission status in a heterogeneous limited disease small-cell lung cancer patient cohort treated with definitive chemoradiotherapy. *BMC cancer*, 16:216, 2016.

[96] A. Ciarrocchi, R. Pietroletti, F. Carlei, and G. Amicucci. Extensive surgery and lymphadenectomy do not improve survival in primary melanoma of the anorectum:

results from analysis of a large database (SEER). *Colorectal Disease*, 19(2):158–164, feb 2017.

[97] Sughosh Dhakal, James E Bates, Carla Casulo, Jonathan W Friedberg, Michael W Becker, Jane L Liesveld, and Louis S Constine. Patterns and Timing of Failure for Diffuse Large B-Cell Lymphoma After Initial Therapy in a Cohort Who Underwent Autologous Bone Marrow Transplantation for Relapse. *International journal of radiation oncology, biology, physics*, 96(2):372–378, 2016.

[98] Ali H.R., Dawson S.-J., Blows F.M., Provenzano E., Leung S., Nielsen T., and Pharoah P.D. A Ki67/BCL2 index based on immunohistochemistry is highly prognostic in ER-positive breast cancer. *Journal of Pathology*, 226(1):97–107, 2012.

[99] Seok Jin Kim, Dok Hyun Yoon, Arnaud Jaccard, Wee Joo Chng, Soon Thye Lim, Huangming Hong, Yong Park, Kian Meng Chang, Yoshinobu Maeda, Fumihiro Ishida, Dong-Yeop Shin, Jin Seok Kim, Seong Hyun Jeong, Deok-Hwan Yang, Jae-Cheol Jo, Gyeong-Won Lee, Chul Won Choi, Won-Sik Lee, Tsai-Yun Chen, Kiyeun Kim, Sin-Ho Jung, Tohru Murayama, Yasuhiro Oki, Ranjana Advani, Francesco D'Amore, Norbert Schmitz, Cheolwon Suh, Ritsuro Suzuki, Yok Lam Kwong, Tong-Yu Lin, and Won Seog Kim. A prognostic index for natural killer cell lymphoma after non-anthracycline-based treatment: a multicentre, retrospective analysis. *The Lancet. Oncology*, 17(3):389–400, 2016.

[100] Icro Meattini, Calogero Saieva, Paolo Bastiani, Francesca Martella, Giulio Francolini, Monica Lo Russo, Lisa Paoletti, Morena Doria, Isacco Desideri, Francesca Terziani, Carla De Luca Cardillo, Benedetta Bendinelli, Cinzia Ciabatti, Cristina Muntoni, Galliano Tinacci, Jacopo Nori, Herd Smith, Beniamino Brancato, Lorenzo Galli, Luis Jose Sanchez, Donato Casella, Marco Bernini, Lorenzo Orzalesi, Giulio Alberto Carta, Simonetta Bianchi, Francesca Rossi, and Lorenzo Livi. Impact of hormonal status on outcome of ductal carcinoma in situ treated with breast-conserving surgery plus radiotherapy: Long-term experience from two large-institutional series. *Breast (Edinburgh, Scotland)*, 33:139–144, 2017.

[101] Piet A van den Brandt and Leo J Schouten. Relationship of tree nut, peanut and peanut butter intake with total and cause-specific mortality: a cohort study and meta-analysis. *International journal of epidemiology*, 44(3):1038–1049, 2015.

[102] Ali H.R., Provenzano E., Dawson S.-J., Blows F.M., Liu B., Shah M., Earl H.M., Poole C.J., Hiller L., Dunn J.A., Bowden S.J., Twelves C., Bartlett J.M.S., Mahmoud S.M.A., Rakha E., Ellis I.O., Liu S., Gao D., Nielsen T.O., and Pharoah P.D.P. Association between CD8+ T-cell infiltration and breast cancer survival in 12 439 patients. *Annals of Oncology*, 25(8):1536–1543, 2014.

[103] Dianed Zheng D., Christ S.L., Lam B.L., Arheart K.L., and Galor A. Increased mortality risk among the visually impaired: The roles of mental well-being and preventive care practices. *Investigative Ophthalmology and Visual Science*, 53(6):2685–2692, 2012.

[104] Elias Jabbour, Guillermo Garcia-Manero, A Megan Cornelison, Jorge E Cortes, Farhad Ravandi, Naval Daver, Tapan Kadia, Angela Teng, and Hagop Kantarjian. The effect of decitabine dose modification and myelosuppression on response and survival in patients with myelodysplastic syndromes. *Leukemia & lymphoma*, 56(2):390–394, 2015.

[105] N A Quraishi, S R Manoharan, G Arealis, A Khurana, S Elsayed, K L Edwards, and B M Boszczyk. Accuracy of the revised Tokuhashi score in predicting survival in patients with metastatic spinal cord compression (MSCC). *European spine journal : official publication of the European Spine Society, the European Spinal Deformity Society, and the European Section of the Cervical Spine Research Society*, 22 Suppl 1:S21–6, 2013.

[106] Rance J.T. Fujiwara, Barbara Burtness, Zain A. Husain, Benjamin L. Judson, Aarti Bhatia, Clarence T. Sasaki, Wendell G. Yarbrough, and Saral Mehra. Treatment guidelines and patterns of care in oral cavity squamous cell carcinoma: Primary surgical resection vs. nonsurgical treatment. *Oral Oncology*, 71:129–137, aug 2017.

[107] Daniel Carlzon, Johan Svensson, Max Petzold, Magnus K Karlsson, Osten Ljunggren, Mohammad-Ali Haghsheno, Jan-Erik Damber, Dan Mellstrom, and Claes Ohlsson. Insulin-like growth factor I and risk of incident cancer in elderly men - results from MrOS (Osteoporotic Fractures in Men) in Sweden. *Clinical endocrinology*, 84(5):764–770, 2016.

[108] Prinelli F., Yannakoulia M., Anastasiou C.A., Adorni F., Di Santo S.G., Musicco M., and Scarmeas N. Mediterranean diet and other lifestyle factors in relation to 20-year all-cause mortality: A cohort study in an Italian population. *British Journal of Nutrition*, 113(6):1003–1011, 2015.

[109] Eugene P Ceppa, Alexandra M Roch, Jessica L Cioffi, Neil Sharma, Jeffrey J Easler, John M DeWitt, Michael G House, Nicholas J Zyromski, Attila Nakeeb, and C Max Schmidt. Invasive, mixed-type intraductal papillary mucinous neoplasm: superior prognosis compared to invasive main-duct intraductal papillary mucinous neoplasm. *Surgery*, 158(4):935–937, 2015.

[110] Naamit K Gerber, Yoshiya Yamada, Andreas Rimner, Weiji Shi, Gregory J Riely, Kathryn Beal, Helena A Yu, Timothy A Chan, Zhigang Zhang, and Abraham J

Wu. Erlotinib versus radiation therapy for brain metastases in patients with EGFR-mutant lung adenocarcinoma. *International journal of radiation oncology, biology, physics*, 89(2):322–329, 2014.

[111] Danielle Rodin, Michael Drumm, Rebecca Clayman, Daniela L. Buscariollo, Sigolene Galland-Girodet, Alec Eidelman, Adam S. Feldman, Douglas M. Dahl, Francis J. McGovern, Aria F. Olumi, Andrzej Niemierko, William U. Shipley, Anthony L. Zietman, and Jason A. Efstathiou. Risk Factors for Disease Progression After Post-prostatectomy Salvage Radiation: Long-term Results of a Single-institution Experience. *Clinical Genitourinary Cancer*, 16(1):21–27.e1, feb 2018.

[112] Kent M.S., Mandrekar S.J., Landreneau R., Nichols F., Foster N.R., Dipetrillo T.A., Meyers B., Heron D.E., Jones D.R., Tan A.D., Starnes S., and Putnam J.B. A Nomogram to Predict Recurrence and Survival of High-Risk Patients Undergoing Sublobar Resection for Lung Cancer: An Analysis of a Multicenter Prospective Study (ACOSOG Z4032). *Annals of Thoracic Surgery*, 102(1):239–246, 2016.

[113] Anthony N Karnezis, Samuel Leung, Jamie Magrill, Melissa K McConechy, Winnie Yang, Christine Chow, Martin Kobel, Cheng-Han Lee, David G Huntsman, Aline Talhouk, Friederich Kommoss, C Blake Gilks, and Jessica N McAlpine. Evaluation of endometrial carcinoma prognostic immunohistochemistry markers in the context of molecular classification. *The Journal of Pathology: Clinical Research*, 3(4):279–293, oct 2017.

[114] Akihiro Naito, Satoru Taguchi, Tohru Nakagawa, Akihiko Matsumoto, Yasushi Nagase, Mariko Tabata, Jimpei Miyakawa, Motofumi Suzuki, Hiroaki Nishimatsu, Yutaka Enomoto, Shintaro Takahashi, Toshikazu Okaneya, Daisuke Yamada, Takamitsu Tachikawa, Shigeru Minowada, Tetsuya Fujimura, Hiroshi Fukuhara, Haruki Kume, and Yukio Homma. Prognostic significance of serum neuron-specific enolase in small cell carcinoma of the urinary bladder. *World journal of urology*, 35(1):97–103, 2017.

[115] Satoru Taguchi, Tohru Nakagawa, Akihiko Matsumoto, Yasushi Nagase, Taketo Kawai, Yoshinori Tanaka, Kanae Yoshida, Sachi Yamamoto, Yutaka Enomoto, Yorito Nose, Toshikazu Sato, Akira Ishikawa, Yukari Uemura, Tetsuya Fujimura, Hiroshi Fukuhara, Haruki Kume, and Yukio Homma. Pretreatment neutrophil-to-lymphocyte ratio as an independent predictor of survival in patients with metastatic urothelial carcinoma: A multi-institutional study. *International journal of urology : official journal of the Japanese Urological Association*, 22(7):638–643, 2015.

[116] Lars Barregard, Gerd Sallsten, Bjorn Fagerberg, Yan Borne, Margaretha Persson, Bo Hedblad, and Gunnar Engstrom. Blood Cadmium Levels and Incident Cardiovascular Events during Follow-up in a Population-Based Cohort of Swedish Adults: The

Malmo Diet and Cancer Study. *Environmental health perspectives*, 124(5):594–600, 2016.

[117] Chen S., Huang L., Liu Y., Chen C.M., and Wu J. The predictive and prognostic significance of pre- and post-treatment topoisomerase IIalpha in anthracycline-based neoadjuvant chemotherapy for local advanced breast cancer. *European Journal of Surgical Oncology*, 39(6):619–626, 2013.

[118] Mantripragada K.C., Hamid F., Shafqat H., Kalyan C Mantripragada, Fatima Hamid, Hammad Shafqat, and Adam J Olszewski. Adjuvant Therapy for Resected Gallbladder Cancer: Analysis of the National Cancer Data Base. *Journal of the National Cancer Institute*, 109(2), 2017.

[119] Randy C Miles, Rachel E Gullerud, Christine M Lohse, James W Jakub, Amy C Degnim, and Judy C Boughey. Local recurrence after breast-conserving surgery: multivariable analysis of risk factors and the impact of young age. *Annals of surgical oncology*, 19(4):1153–1159, 2012.

[120] Leu S., Von Felten S., Frank S., Vassella E., Vajtai I., Taylor E., Schulz M., Hutter G., Hench J., Schucht P., Boulay J.-L., Severina Leu, Stefanie von Felten, Stephan Frank, Erik Vassella, Istvan Vajtai, Elisabeth Taylor, Marianne Schulz, Gregor Hutter, Jurgen Hench, Philippe Schucht, Jean-Louis Boulay, and Luigi Mariani. IDH/MGMT-driven molecular classification of low-grade glioma is a strong predictor for long-term survival. *Neuro-Oncology*, 15(4):469–479, 2013.

[121] Joost C. de Vries, Berdien Oortgiesen, Marc H. Hemmelder, Eric van Roon, Robby E. Kibbelaar, Nic Veeger, and Mels Hoogendoorn. Restoration of renal function in patients with newly diagnosed multiple myeloma is not associated with improved survival: a population-based study. *Leukemia & Lymphoma*, 58(9):2101–2109, sep 2017.

[122] Fradet V., Mauermann J., Kassouf W., Rendon R., Jacobsen N., Fairey A., Izawa J., Kapoor A., Black P., Tanguay S., Chin J., So A., Lattouf J.-B., Bell D., Saad F., Sheyegan B., Drachenberg D., and Cagiannos I. Risk factors for bladder cancer recurrence after nephroureterectomy for upper tract urothelial tumors: Results from the Canadian Upper Tract Collaboration. *Urologic Oncology: Seminars and Original Investigations*, 32(6):839–845, 2014.

[123] G David Batty, Tom C Russ, Emmanuel Stamatakis, and Mika Kivimaki. Psychological distress in relation to site specific cancer mortality: Pooling of unpublished data from 16 prospective cohort studies. *BMJ (Online)*, 356:j108, 2017.

[124] Waqas R Shaikh, Stephen W Dusza, Martin A Weinstock, Susan A Oliveria, Alan C Geller, and Allan C Halpern. Melanoma Thickness and Survival Trends in the United States, 1989 to 2009. *Journal of the National Cancer Institute*, 108(1), jan 2016.

[125] Schmidt N., Hess V., Zumbrunn T., Rothermundt C., and Bongartz G. Choi response criteria for prediction of survival in patients with metastatic renal cell carcinoma treated with anti-angiogenic therapies. *European Radiology*, 23(3):632–639, 2013.

[126] Sara Alonso-Alvarez, Laura Magnano, Miguel Alcoceba, Marcio Andrade-Campos, Natalia Espinosa-Lara, Guillermo Rodriguez, Santiago Mercadal, Itziar Carro, Juan M Sancho, Miriam Moreno, Antonio Salar, Francesc Garcia-Pallarols, Reyes Arranz, Jimena Cannata, Maria Jose Terol, Ana I Teruel, Antonia Rodriguez, Ana Jimenez-Ubieto, Sonia Gonzalez de Villambrosia, Jose L Bello, Lourdes Lopez, Silvia Monsalvo, Silvana Novelli, Erik de Cabo, Maria S Infante, Emilia Pardal, Maria Garcia-Alvarez, Julio Delgado, Marcos Gonzalez, Alejandro Martin, Armando Lopez-Guillermo, and Maria D Caballero. Risk of, and survival following, histological transformation in follicular lymphoma in the rituximab era. A retrospective multicentre study by the Spanish GELTAMO group. *British journal of haematology*, 178(5):699–708, 2017.

[127] Palak J Trivedi, Willem J Lammers, Henk R van Buuren, Albert Pares, Annarosa Floreani, Harry L A Janssen, Pietro Invernizzi, Pier Maria Battezzati, Cyriel Y Ponsioen, Christophe Corpechot, Raoul Poupon, Marlyn J Mayo, Andrew K Burroughs, Frederik Nevens, Andrew L Mason, Kris V Kowdley, Ana Lleo, Llorenc Caballeria, Keith D Lindor, Bettina E Hansen, Gideon M Hirschfield, and Global P B C Study Group. Stratification of hepatocellular carcinoma risk in primary biliary cirrhosis: a multicentre international study. *Gut*, 65(2):321–329, 2016.

[128] Malte W Vetterlein, Philipp Gild, Luis A Kluth, Thomas Seisen, Michael Gierth, Hans-Martin Fritsche, Maximilian Burger, Chris Protzel, Oliver W Hakenberg, Nicolas von Landenberg, Florian Roghmann, Joachim Noldus, Philipp Nuhn, Armin Pycha, Michael Rink, Felix K-H Chun, Matthias May, Margit Fisch, Atiqullah Aziz, and PROMETRICS 2011 Study Group. Peri-operative allogeneic blood transfusion does not adversely affect oncological outcomes after radical cystectomy for urinary bladder cancer: a propensity score-weighted European multicentre study. *BJU international*, 121(1):101–110, 2018.

[129] Peters M., van der Voort van Zyp J.R.N., Moerland M.A., Hoekstra C.J., van de Pol S., Westendorp H., Maenhout M., Kattevilder R., Verkooijen H.M., van Rossum P.S.N., Ahmed H.U., Shah T.T., Emberton M., and van Vulpen M A O Peters M.; ORCID: http://orcid.org/0000-0002-7981-5768 A O Westendorp H.; ORCID: http://orcid.org/0000-0001-9549-2391 A O Shah T.T.; ORCID:

Http://orcid.org/0000-0003-1642-1208. Development and internal validation of a multivariable prediction model for biochemical failure after whole-gland salvage iodine-125 prostate brachytherapy for recurrent prostate cancer. *Brachytherapy*, 15(3):296–305, 2016.

[130] Mette Calundann Noer, Pia Leandersson, Torbjørn Paulsen, Susanne Rosthøj, Sofie Leisby Antonsen, Christer Borgfeldt, and Claus Høgdall. Confounders other than comorbidity explain survival differences in Danish and Swedish ovarian cancer patients – a comparative cohort study. *Acta Oncologica*, pages 1–9, feb 2018.

[131] Nathan Papa, Nathan Lawrentschuk, David Muller, Robert MacInnis, Anthony Ta, Gianluca Severi, Jeremy Millar, Rodney Syme, Graham Giles, and Damien Bolton. Rural residency and prostate cancer specific mortality: results from the Victorian Radical Prostatectomy Register. *Australian and New Zealand journal of public health*, 38(5):449–454, 2014.

[132] Clive A.O., Kahan B.C., Hooper C.E., Bhatnagar R., Morley A.J., Zahan-Evans N., Bintcliffe O.J., Boshuizen R.C., Fysh E.T.H., Tobin C.L., Medford A.R.L., Harvey J.E., Van Den Heuvel M.M., and Lee Y.C.G. Predicting survival in malignant pleural effusion: Development and validation of the LENT prognostic score. *Thorax*, 69(12):1098–1104, 2014.

[133] Shanna A. Arnold Egloff, Liping Du, Holli A. Loomans, Alina Starchenko, Pei-Fang Su, Tatiana Ketova, Paul B. Knoll, Jifeng Wang, Ahmed Q. Haddad, Oluwole Fadare, Justin M. Cates, Yair Lotan, Yu Shyr, Peter E. Clark, and Andries Zijlstra. Shed urinary ALCAM is an independent prognostic biomarker of three-year overall survival after cystectomy in patients with bladder cancer. *Oncotarget*, 8(1), jan 2017.

[134] Riedel D.J., Cox E.R., and Stafford K.A. Clinical presentation and outcomes of prostate cancer in an urban cohort of predominantly African American, human immunodeficiency virus-infected patients. *Urology*, 85(2):415–421, 2015.

[135] M C van Maaren, L de Munck, J J Jobsen, P Poortmans, G H de Bock, S Siesling, and L J A Strobbe. Breast-conserving therapy versus mastectomy in T1-2N2 stage breast cancer: a population-based study on 10-year overall, relative, and distant metastasis-free survival in 3071 patients. *Breast cancer research and treatment*, 160(3):511–521, 2016.

[136] Paul W Sperduto, Norbert Kased, David Roberge, Samuel T Chao, Ryan Shanley, Xianghua Luo, Penny K Sneed, John Suh, Robert J Weil, Ashley W Jensen, Paul D Brown, Helen A Shih, John Kirkpatrick, Laurie E Gaspar, John B Fiveash, Veronica Chiang, Jonathan P S Knisely, Christina Maria Sperduto, Nancy Lin, and Minesh

Mehta. The effect of tumor subtype on the time from primary diagnosis to development of brain metastases and survival in patients with breast cancer. *Journal of neuro-oncology*, 112(3):467–472, 2013.

[137] Cata J.P., Jones J., Sepesi B., Mehran R.J., Rodriguez-Restrepo A., Lasala J., and Feng L. Lack of Association Between Dexamethasone and Long-Term Survival After Non-Small Cell Lung Cancer Surgery. *Journal of Cardiothoracic and Vascular Anesthesia*, 30(4):930–935, 2016.

[138] Johann von Felden, Denise Heim, Kornelius Schulze, Till Krech, Florian Ewald, Bjorn Nashan, Ansgar W Lohse, and Henning Wege. High expression of micro RNA-135A in hepatocellular carcinoma is associated with recurrence within 12 months after resection. *BMC cancer*, 17(1):60, 2017.

[139] Malte W. Vetterlein, Julia Roschinski, Philipp Gild, Phillip Marks, Armin Soave, Ousman Doh, Hendrik Isbarn, Wolfgang Höppner, Walter Wagner, Shahrokh F. Shariat, Maurizio Brausi, Franziska Büscheck, Guido Sauter, Margit Fisch, and Michael Rink. Impact of the Ki-67 labeling index and p53 expression status on disease-free survival in pT1 urothelial carcinoma of the bladder. *Translational Andrology and Urology*, 6(6):1018–1026, dec 2017.

[140] Nemelc R.M., Stadhouder A., and van Royen B.J. The outcome and survival of palliative surgery in thoraco-lumbar spinal metastases: contemporary retrospective cohort study. *European Spine Journal*, 2014.

[141] Ishimaru M., Ono S., Suzuki S., Matsui H., Fushimi K., Miho; ORCID: http://orcid.org/0000 0002-5269-5698 Yasunaga H. AO - Ishimaru, Miho Ishimaru, Sachiko Ono, Sayaka Suzuki, Hiroki Matsui, Kiyohide Fushimi, and Hideo Yasunaga. Risk Factors for Free Flap Failure in 2,846 Patients With Head and Neck Cancer: A National Database Study in Japan. *Journal of oral and maxillofacial surgery : official journal of the American Association of Oral and Maxillofacial Surgeons*, 74(6):1265–1270, 2016.

[142] Hsia T.E.-C., Tu C.-Y., Chen H.-J., Chen S.-C., Liang J.I.-A.N., Chen C.-Y.I., and Wang Y.-C. A population-based study of primary chemoradiotherapy in clinical stage III non-small cell lung cancer: Intensity-modulated radiotherapy versus 3D conformal radiotherapy. *Anticancer Research*, 34(9):5175–5180, 2014.

[143] Anna Ehinger, Per Malmström, Pär-Ola Bendahl, Christopher W Elston, Anna-Karin Falck, Carina Forsare, Dorthe Grabau, Lisa Rydén, Olle Stål, Mårten Fernö, and South and South-East Swedish Breast Cancer Groups. Histological grade provides significant prognostic information in addition to breast cancer subtypes defined according to St Gallen 2013. *Acta oncologica (Stockholm, Sweden)*, 56(1):68–74, jan 2017.

[144] Wasil Jastaniah, Naglla Elimam, Razan S Alluhaibi, Alaa T Alharbi, Adil Ah Abbas, and Mohammed B Abrar. The prognostic significance of hypertension at diagnosis in children with wilms tumor. *Saudi medical journal*, 38(3):262–267, 2017.

[145] Rachel S van der Post, Ingrid P Vogelaar, Peggy Manders, Lizet E van der Kolk, Annemieke Cats, Liselotte P van Hest, Rolf Sijmons, Cora M Aalfs, Margreet G E M Ausems, Encarna B Gomez Garcia, Anja Wagner, Frederik J Hes, Neeltje Arts, Arjen R Mensenkamp, J Han van Krieken, Nicoline Hoogerbrugge, and Marjolijn J L Ligtenberg. Accuracy of Hereditary Diffuse Gastric Cancer Testing Criteria and Outcomes in Patients With a Germline Mutation in CDH1. *Gastroenterology*, 149(4):897–906.e19, 2015.

[146] Yu E., Stitt L., Vujovic O., Joseph K., Assouline A., Au J., Younus J., and Perera F. Prognostic factors for male breast cancer: Similarity to female counterparts. *Anticancer Research*, 33(5):2227–2232, 2013.

[147] Rusthoven C.G., Koshy M., Sher D.J., Ney D.E., Gaspar L.E., Jones B.L., Karam S.D., Amini A., Ormond D.R., and Youssef A.S. Combined-modality therapy with radiation and chemotherapy for elderly patients with glioblastoma in the temozolomide era: A national cancer database analysis. *JAMA Neurology*, 73(7):821–828, 2016.

[148] Laura E Hudson, Shishir K Maithel, Grant W Carlson, Monica Rizzo, Douglas R Murray, Andrea C Hestley, and Keith A Delman. 1 or 2 cm margins of excision for T2 melanomas: do they impact recurrence or survival?. *Annals of surgical oncology*, 20(1):346–351, 2013.
